# Supplementary material for: Strain stiffening of Ndc80 complexes attached to microtubule plus ends
Source: Biophys J. 2022 Oct 4;121(21):4048–62. doi: 10.1016/j.bpj.2022.09.039 (PMC9675032; doi:10.1016/j.bpj.2022.09.039)
Supplement: Document S2. Article plus supporting material [file mmc2.pdf]

Biophysical Journal
Article

Biophysical Society

Strain stiffening of Ndc80 complexes attached to microtubule plus ends

Felix Schwietert,[1] Vladimir A. Volkov,[2,3] Pim J. Huis in 't Veld,[4] Marileen Dogterom,[3] Andrea Musacchio,[4] and Jan Kierfeld[1,*]

[1]Physics Department, TU Dortmund University, Dortmund, Germany; [2]School of Biological and Behavioural Sciences, Queen Mary University of London, London, UK; [3]Department of Bionanoscience, Faculty of Applied Sciences, Delft University of Technology, Delft, Netherlands; and [4]Department of Mechanistic Cell Biology, Max Planck Institute of Molecular Physiology, Dortmund, Germany

ABSTRACT In the mitotic spindle, microtubules attach to chromosomes via kinetochores. The microtubule-binding Ndc80 complex is an integral part of kinetochores, and is essential for kinetochores to attach to microtubules and to transmit forces from dynamic microtubule ends to the chromosomes. The Ndc80 complex has a rod-like appearance with globular domains at its ends that are separated by a long coiled coil. Its mechanical properties are considered important for the dynamic interaction between kinetochores and microtubules. Here, we present a novel method that allows us to time trace the effective stiffness of Ndc80 complexes following shortening microtubule ends against applied force in optical trap experiments. Applying this method to wild-type Ndc80 and three variants (calponin homology (CH) domains mutated or Hec1 tail unphosphorylated, phosphorylated, or truncated), we reveal that each variant exhibits strain stiffening; i.e., the effective stiffness increases under tension that is built up by a depolymerizing microtubule. The strain stiffening relation is roughly linear and independent of the state of the microtubule. We introduce structure-based models that show that the strain stiffening can be traced back to the specific architecture of the Ndc80 complex with a characteristic flexible kink, to thermal fluctuations of the microtubule, and to the bending elasticity of flaring protofilaments, which exert force to move the Ndc80 complexes. Our model accounts for changes in the amount of load-bearing attachments at various force levels and reproduces the roughly linear strain stiffening behavior, highlighting the importance of force-dependent binding affinity.

SIGNIFICANCE By time-tracing the stiffness of microtubule end-tracking Ndc80 complexes in optical trap experiments, we detect strain stiffening, and, thereby, provide new insights into the elastic properties of the Ndc80 complex. The strain stiffening is robust against mutations in the Ndc80 complex. We relate strain stiffening to the structure of the Ndc80 complex by means of a simple polymer model, to thermal fluctuations of the microtubule, and to the flexibility of force-generating flaring protofilaments at the tip of the microtubule. Since Ndc80 complexes play a major role in transmitting force from microtubule ends to the kinetochore, their elastic properties are of great interest for a deeper understanding of chromosome dynamics in the mitotic spindle.

INTRODUCTION

In the mitotic spindle, microtubules (MTs) attach to chromosomes via macromolecular structures called kinetochores (1). Kinetochores bind to MTs via attachments created by Ndc80 complexes (2). The Ndc80-mediated attachments remain intact while dynamic MTs alternate between growth and shrinkage, and transmit depolymerization forces to the kinetochore during the segregation of chromosomes. The exact mechanisms underlying binding and force transmission are not completely understood but are expected to reflect the molecular structure and resulting elastic properties of the Ndc80 complex.

The Ndc80 complex is a four-subunit rod-like coiled coil with a characteristic flexible kink at approximately one-third of its length (3,4). MT binding is performed by the globular calponin homology (CH) domains near the N-terminal ends of the Ndc80 and Nuf2 subunits, and an unstructured N-terminal tail. In vitro experiments reveal that a single Ndc80 complex is not able to track dynamic MT ends (5–7), whereas tracking activity is attained by multimerization (5,7) and in the presence of Dam1 or Ska complexes (6,8,9). Moreover, an unstructured positively charged Ndc80 tail that precedes the CH domain is crucial

Submitted March 15, 2022, and accepted for publication August 27, 2022.

*Correspondence: jan.kierfeld@tu-dortmund.de

Editor: Kristen Verhey.

https://doi.org/10.1016/j.bpj.2022.09.039

© 2022 Biophysical Society.

This is an open access article under the CC BY license (http://creativecommons.org/licenses/by/4.0/).

for MT tip tracking (10), and phosphorylation of the tail or introduction of negative charges reduces the binding affinity of the Ndc80 complex to the MT lattice (2,11–14).

Early theoretical models of spindle-kinetochore dynamics envisioned a static Hill sleeve with rigid linkers (15,16) or motor proteins mediating MT-kinetochore attachment and movement (17). More recent studies included (visco)elastic MT-kinetochore linkers with a constant stiffness, which had to be estimated (18–21). One such model identified the linker stiffness as a critical parameter for the occurrence of chromosome oscillations during metaphase (22). With the identification of the Ndc80 complex as the crucial linker, understanding its elastic properties has now become crucially important to dissect how MT depolymerization forces are transmitted to the kinetochore to generate movement. The elastic properties of the Ndc80 complex in its MT-bound state, in particular its stiffness under tension, are the main subject of this work.

Measurements of stiffness can confirm structural models if they are in agreement with elastic models derived from the available structural information on the Ndc80 complex. The elastic properties of the Ndc80 complex were studied experimentally by optical trapping methods introduced in (7). Here, we re-analyzed them using novel theoretical analysis and modeling approaches. In those experiments, the stiffness of Ndc80 complexes was found to increase under tension (7). Although in those experiments the stiffness was only determined while the Ndc80 complexes were attached to a stalled MT, here, we re-evaluate the same data with a novel method that allows us to time trace the stiffness; i.e., to determine the stiffness of Ndc80 complexes while it tracks a polymerizing or depolymerizing MT against the opposing load from the optical trap. By splitting the force traces into fixed time intervals and analyzing stiffness at each of them, we achieve several advantages: 1) we generate more data over a wider force range; 2) we demonstrate stiffening during force production and not only compare stalled MTs to free ones; 3) we alleviate the concern that different levels of strain stiffening result from differences in MT ends or beads. Time tracing also allows us to establish that strain stiffening does not depend on the state of the MT end: we observe positive correlation of stiffness with force when the MT stalls, grows, or shrinks. In addition to wild-type Ndc80, we also analyze data from MT end-tracking experiments with Ndc80 complexes whose tails are phosphorylated or truncated (10), or harboring CH domains that are mutated to greatly reduce MT binding. We observe strain stiffening for each of those Ndc80 mutations and draw conclusions on the role of the tail domain in MT binding.

In order to rationalize the experimental findings on strain stiffening of the Ndc80 links, we introduce a simple polymer model for the Ndc80 complex. Describing Ndc80 as an ideal chain with two bonds of different lengths, we show that the strain stiffening is a direct consequence of the characteristic Ndc80 structure with its two stiff rods that are connected flexibly. To match the measured stiffnesses, it is necessary to include the contributions from MT fluctuations and from protofilament (PF) bending to the total effective stiffness. Finally, to explain the shape of the experimental stiffness-force relations, we also introduce a positive correlation of the number of MT end-attached Ndc80 complexes with MT-generated force.

MATERIALS AND METHODS

Experimental methods

Cloning and purification

The Ndc80 CH-mutant contains Hec1 K89A, Hec1 K166A, Nuf2 K41A, and Nuf2 K115A: four mutations that reduce the binding of Ndc80 to MTs (23). Mutations were introduced into pLIB-Hec1 and pLIB-Nuf2 vectors using site-directed mutagenesis and Gibson assembly. Expression cassettes containing mutated Hec1, mutated Nuf2, Spc25-Sortase-Hexahistide, and Spc24-Spy were combined on a pBIG1 plasmid and integrated into a single baculovirus for expression in insect cells (24). The Ndc80 CH-mutant was purified and linked to T_1S_3 modules (25) as previously described in detail for wild type, tailless, and phosphorylated Ndc80 variants (7,10).

Optical trap experiments

Optical trap experiments with dynamic MTs and Ndc80 were performed as described previously (7,10). In brief, 1-μm silica beads were coated with poly-l-lysine-grafted polyethylene glycol (PLL-PEG) as described previously (7). Between 1% and 10% of the PLL-PEG carried a biotin, and was subsequently saturated with T_1S_3 $[Ndc80]_3$ modules. Ndc80-TMR fluorescence on the bead was measured to determine the coating density, and bead preparations containing on average 100–1000 Ndc80 copies per bead were used for optical trapping.

The microscopic flow chambers were assembled using glass slides and coverslips treated with 2% dichlorodimethylsilane. The chambers were incubated with 0.2 μM anti-digoxigenin immunoglobulin (Ig) G (Roche). Non-specific binding of molecules to coverslips was prevented by an incubation with 1% Pluronic F-127. Then, GMPCPP-stabilized, digoxigenin-labeled MT seeds were attached to the coverslips. Finally, T_1S_3 $[Ndc80]_3$-coated beads were introduced in a reaction mix containing 10–12 μM tubulin, 1 mM GTP, 1 mg/mL κ-casein, 4 mM DTT, 0.2 mg/mL catalase, 0.4 mg/mL glucose oxidase, and 20 mM glucose.

Optical trapping experiments were performed as described previously (7). Before each experiment, a free bead was attached to an MT near its growing end and held in an optical trap with a stiffness of 0.02–0.04 pN nm^{-1}. Position of the bead was continuously recorded at 10 kHz using a quadrant detector, and differential interference contrast (DIC) images were simultaneously recorded to monitor positions of the bead and the dynamics of MTs. The bead was held in a trap until the MT depolymerized, which resulted in two alternative outcomes: the bead-MT connection broke, or the bead rescued the MT shortening, and the MT started regrowing. In case of a rescue, recordings were stopped arbitrarily after 30 or more minutes by increasing the trap stiffness to 0.2–0.4 pN nm^{-1} and rupturing the bead-MT connection. Quadrant detector readings of the bead position were later turned so that one of the axes corresponded to bead displacement along MT to generate time traces $x(t)$ of the bead position.

Time-tracing the stiffness

As long as the mean bead position is stationary, e.g., during MT stall, the effective stiffness can be easily calculated from the variance of the bead position x by $k_BT/\mathrm{Var}(x)$. We now present a method that enables us to monitor

the stiffness over time, i.e., also during polymerization and depolymerization phases of the MT, and to observe strain stiffening of the Ndc80 complex in the course of a single experiment. We divide the signal into several intervals k of a certain length Δt for each of which we will determine a stiffness as sketched in Fig. 1. For that purpose, we do not use the variance over that interval but the mean squared distance from a quasi-equilibrium bead position, which may slowly vary over time. We estimate the time course of the quasi-equilibrium bead position with a linear fit $f_k(t)$ to the entire time trace $x_k(t)$ in an interval k. Then, the effective stiffness during the interval k is given by the inverse of the mean squared distance

$$c_k = \frac{k_B T}{\langle (x_k(t) - f_k(t))^2 \rangle}. \tag{1}$$

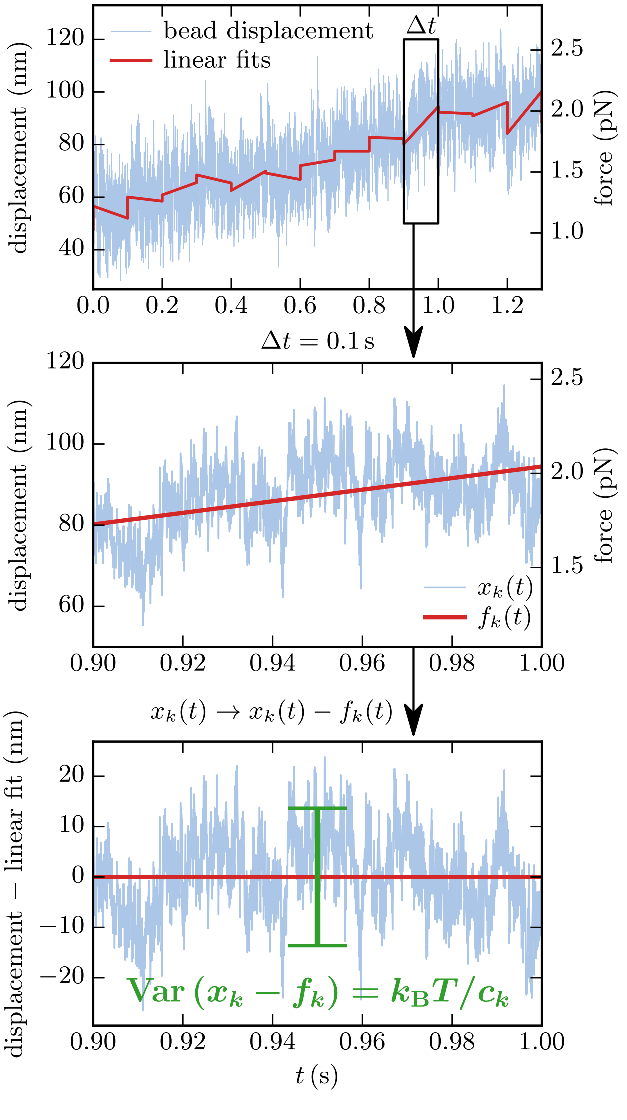

FIGURE 1 Stiffness time-tracing. The signal is divided in intervals of 0.1 s, which gives 1000 data points per interval at a sampling rate of 10 kHz. For each interval, we estimate the evolution of the equilibrium bead position with a linear fit $f_k(t)$. The stiffness of an interval is then given by Eq. 1, where the variance equals the mean squared distance of the bead position $x_k(t)$ from the estimated equilibrium $f_k(t)$. To see this figure in color, go online.

Finally, we can assign a force F_k on the Ndc80 link to each interval, which can be calculated from the trap stiffness and the mean bead position during that interval, $F_k = c_{trap}\langle x_k \rangle$.

The time interval Δt should be sufficiently small to describe a time-resolved stiffness but, at the same time, should cover enough independent displacement measurements for a trustful variance determination. Typical autocorrelation times are $\gamma / c \sim 0.1$–1 ms, where γ is the friction coefficient and c the stiffness. For our experiments, $\Delta t = 0.1$ s has proved to be a good choice, which results in 100–1000 independent measurements within each interval.

Theoretical model

Before modeling the outcomes of the stiffness measurements, we have to identify the elastic elements that contribute to the total stiffness as sketched in Fig. 2 *B*. The stiffness of the optical trap will always be present in the measurements. Its value is determined during the calibration of the optical trap after the bead detached from the MT.

Further potentially elastic elements connect the bead and the MT seed that is fixed on the coverslip. On the one hand, this is the MT, which can exhibit both a mechanical (stretching or bending) and an entropic stiffness (from thermal fluctuations). On the other hand, the link between the MT and the bead has elastic properties. We attribute the elasticity of the link to its four constituents: first, the Ndc80 complex can act as an entropic spring with stiffness c_{Ndc} due to its flexible kink; second, stretching the flaring ends of the PFs at the MT tip out of their preferred curvature may produce an effective stiffness c_{PF} for pulling in the axial direction of the MT; third, the bond between the Ndc80 trimer and the MT may also exhibit some effective elasticity c_{bond}, e.g., as a consequence of unbinding and rebinding of individual Ndc80 units; and, finally, the PLL-PEG that connects the Ndc80 complexes with the bead is a flexible polymeric linker with an entropic stiffness c_{PEG}.

Each of these four stiffnesses may depend on the applied force and exhibit strain stiffening itself. Since the four elements are aligned in series, their inverse stiffnesses sum up to the inverse linker stiffness $\tilde{c}^{-1}$:

$$\tilde{c}(F) = \left(\frac{1}{c_{PEG}(F)} + \frac{1}{c_{Ndc}(F)} + \frac{1}{c_{bond}(F)} + \frac{1}{c_{PF}(F)} \right)^{-1}. \tag{2}$$

It is important to note that this reduces the total stiffness and that the softest elastic element dominates the total stiffness.

It is possible that multiple Ndc80 complexes are attached parallelly to the MT. For the sake of simplicity, we will always assume that each PF can only attach one Ndc80 complex and that the parallel PFs and Ndc80 complexes have the same elongation, respectively. Then, the force F is shared equally between the parallel Ndc80-PF units with stiffness $\tilde{c}(F)$, so that the resultant stiffness of n parallelly attached Ndc80 complexes can be written as

$$\tilde{c}_n = n\, \tilde{c}\left(\frac{F}{n}\right). \tag{3}$$

Although parallel stiffnesses add up, the shared force can give rise to an overall reduction $\tilde{c}_n(F) < \tilde{c}(F)$ of the cooperative stiffness if the individual stiffness exhibits strain stiffening $c(F) \propto F^m$ with an exponent $m > 1$. We will find below that such strain stiffening behavior is realized both for Ndc80 and PF stiffness.

The n parallel linkers $\tilde{c}$ are in series with a single MT with stiffness c_{MT}, and the optical trap, whose force is applied on the bead from the opposite side, acts as a parallel spring (see Fig. 2 *A* and *B*). This results in a total stiffness

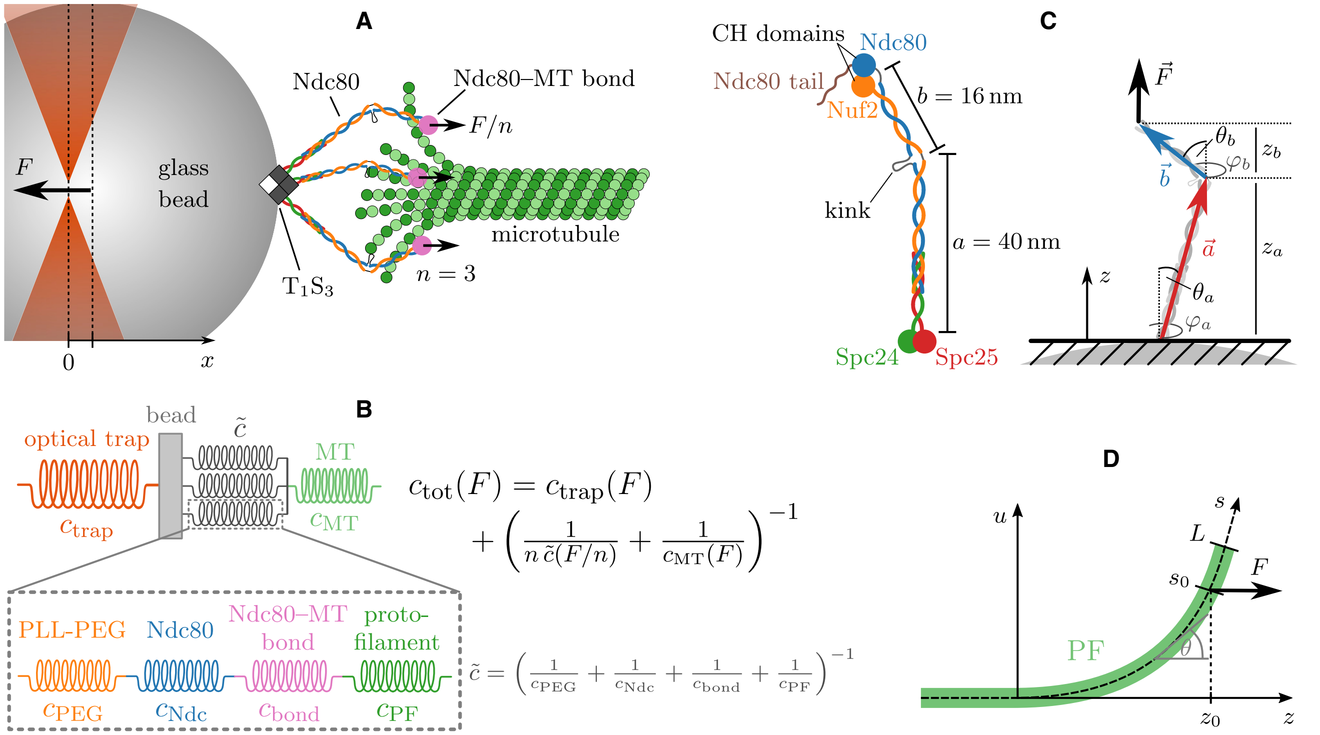

FIGURE 2 (*A*) Sketch of the experiment (not true to scale) with $n = 3$ attached Ndc80 complexes. We assume that each Ndc80 complex is exposed to the same force F/n. (*B*) Spring model. Between the bead and the MT, there are four potentially elastic objects, which are aligned in series: the PLL-PEG, the Ndc80 complex, the Ndc80-MT bond and the curved PF. When n Ndc80 complexes are attached to the MT, n of those serial combinations act as parallel springs. (*C*) Ndc80 complex as FJC. The Ndc80 complex is a tetramer with a total length of 56 nm and a flexible kink at 40 nm. The long arm a is bound to the glass bead, the short arm b to the MT. We model the Ndc80 complex as an FJC with two bonds $\vec{a}$ and $\vec{b}$, which have the different lengths a and b. (*D*) Model for PF bending. The z axis marks the direction along the MT, s is the position along the curved PF. The Ndc80 complex is attached at $s = s_0$ and applies the force F in z direction. To see this figure in color, go online.

$$c_{\text{tot}}(F) = c_{\text{trap}}(F) + \left(\frac{1}{n\,\tilde{c}(F/n)} + \frac{1}{c_{\text{MT}}(F)}\right)^{-1}. \quad (4)$$

We note that the force dependence of the trap stiffness is negligible in the examined force ranges so that we can assume $c_{\text{trap}}(F) = \text{const}$ in the following.

MT stiffness

In the experiments, typical MT lengths L (i.e., the distances between the fixed MT seed and the MT tip linked to the bead) lay in a range between 3 and 10 nm. Given the values of 0.1–2 GPa for the Young's modulus Y of an MT (26), the stiffness for MT stretching can be estimated as $YA/L = 5$ to 330 pN nm^{-1}, which is stiff enough to be ignored in the following. Since the MT tip is lifted from the coverslip and therefore slightly bent when it is bound to the bead, a horizontal force might not only stretch but also unbend the MT. The mechanical stiffness from this unbending can also be neglected, as we show in the Supporting material.

Apart from purely mechanical elasticity, the MT can also exhibit an entropic stiffness, which follows from a description as a semiflexible polymer with thermally excited bending fluctuations. Then, for large forces ($F \gg k_B T/L_p \sim 10^{-6}$ *pN*), the relation between the applied force F and the mean extension z in force direction is given by (27,28)

$$\frac{z}{L} = 1 - \left(\frac{k_B T}{4FL_p}\right)^{\frac{1}{2}}, \quad (5)$$

with the persistence length L_p, which depends on the MT length (29): $L_p = L_p^{\infty}/(1 + L_0^2/L^2)$ with $L_p^{\infty} = 6.3$ mm and $L_0 = 21$ μm. The entropic MT stiffness c_{MT} can be deduced from the derivative of z with respect to the force:

$$\begin{aligned} c_{\text{MT}}(F) &= \left(\frac{\partial z}{\partial F}\right)^{-1} = \frac{4}{L}\left(\frac{L_p}{k_B T}\right)^{\frac{1}{2}} F^{\frac{3}{2}} \\ &= \frac{4}{\sqrt{k_B T}}\left(\frac{L_p^{\infty}}{L^2 + L_0^2}\right)^{\frac{1}{2}} F^{\frac{3}{2}}, \end{aligned} \quad (6)$$

where the length-dependent persistence length was used in the last step.

Bond stiffness

The bond between individual Ndc80 complexes and the MT is established by the globular regions at the N terminus of the complex (3,23,30) via CH domains (31). Also the N-terminal tail of the Ndc80 subunit could be involved in MT binding (1,32). The effective stiffness of the Ndc80-MT bond is hard to model because the exact binding mechanism to the MT is still elusive. Because the Ndc80 CH domain and the N-terminal tail are relevant for binding to the MT, investigation of the stiffness changes for mutants Ndc80$^{\Delta 80}$ and Ndc80^{CHmut} compared with the wild-type Ndc80^{wt} will allow us to address this issue in the "discussion" section. Regarding the linker stiffness $\tilde{c}$, we will first concentrate on the contributions of the Ndc80 complex itself, the PF and the PLL-PEG in the following.

Ndc80 stiffness

In order to model the stiffness of the Ndc80 complex under force, we use a simple polymer model that is based on the known structure (3,4): The Ndc80 complex is a tetramer with a total length of 56 nm and consists of two stiff arms with lengths $a = 40$ nm and $b = 16$ nm, see Fig. 2 *C*. In the cell, the long arm is bound to the kinetochore, in the experiment it is bound to the glass bead, while the short arm can attach to an MT. We describe the conformation of the two arms by two vectors $\vec{a}$ and $\vec{b}$ in polar coordinates as shown in Fig. 2 *C*:

$$\vec{a} = a\begin{pmatrix}\sin\theta_a\cos\varphi_a\\ \sin\theta_a\sin\varphi_a\\ \cos\theta_a\end{pmatrix},\quad \vec{b} = b\begin{pmatrix}\sin\theta_b\cos\varphi_b\\ \sin\theta_b\sin\varphi_b\\ \cos\theta_b\end{pmatrix}. \quad (7)$$

The impenetrable glass bead restricts these two vectors in the experiment. Since the diameter of the glass bead (1 μm) is much larger than the Ndc80 complex, we approximate the bead as a plane surface that confines the Ndc80 complex to the upper half-space, so that $z_a > 0$ and $z = z_a + z_b > 0$ (see Fig. 2 *C*). Experiments have shown that the two arms are connected flexibly within an angular range between 0° ($\vec{a}$ and $\vec{b}$ in line) and 120° (maximally kinked) (4). Since we want to model the Ndc80 complex while it is confined by the glass bead and under the influence of an external stretching force that is applied in the z direction and favors small angles, we can neglect the constraint to angles below 120°. This allows us to perform an explicit analytical calculation, as we reduce the Ndc80 complex to a purely entropic freely jointed chain (FJC) with two bonds of different lengths. The only energy is the stretching energy from a constant external force $\vec{F} = F\vec{e}_z$ in positive z direction,

$$\begin{aligned} E_{\mathrm{FJC}} &= -\vec{F}\cdot\left(\vec{a}+\vec{b}\right) = -F(z_a+z_b) \\ &= -F(a\cos\theta_a + b\cos\theta_b). \end{aligned} \quad (8)$$

Respecting the confinement to the upper half-space, we obtain the canonical partition function ($\beta \equiv 1/k_{\mathrm{B}}T$):

$$Z(F) = \iint_{z_a, z > 0} \mathrm{d}\theta_a \sin\theta_a \mathrm{d}\theta_b \sin\theta_b\, \mathrm{e}^{-\beta E_{\mathrm{FJC}}}. \quad (9)$$

Both the extension-force relation $z(F)$ and the stiffness-force relation $c_{\mathrm{Ndc}}(F)$ in the presence of thermal fluctuations can be obtained from derivatives of the partition function with respect to the force:

$$z(F) = \langle z\rangle = \frac{1}{\beta}\partial_F \ln Z, c_{\mathrm{Ndc}}(F) = \left(\frac{\partial z(F)}{\partial F}\right)^{-1} \quad (10)$$

The explicit result for the stiffness of the Ndc80 complex is

$$c_{\mathrm{Ndc}}(F) = \left[\frac{2}{\beta F^2} + \beta\frac{2\mathrm{e}^{\beta Fa}\left((a^2+b^2)\sinh\beta Fb + 2ab\cosh\beta Fb\right) - b^2\mathrm{e}^{\beta Fb}}{2\mathrm{e}^{\beta Fa}\sinh\beta Fb - \mathrm{e}^{\beta Fb} - \beta Fb + 1} - \beta\left(\frac{2\mathrm{e}^{\beta Fa}(a\sinh\beta Fb + b\cosh\beta Fb) - b\mathrm{e}^{\beta Fb} - b}{2\mathrm{e}^{\beta Fa}\sinh\beta Fb - \mathrm{e}^{\beta Fb} - \beta Fb + 1}\right)^2\right]^{-1}. \quad (11)$$

In the limit of strong forces ($F \gg 1/\beta b = 0.256$ pN), this simplifies to

$$z(F) = a + b - \frac{2}{\beta F}, c_{\mathrm{Ndc}} = \frac{\beta F^2}{2}, \quad (12)$$

which is a general result for arbitrary FJC polymer models under force, where the relative extension of an FJC of total length L and bond length b saturates according to $1 - z(F)/L \propto k_B T/bF$ (28,33).

Protofilament stiffness

We assign a stiffness to the PF by modeling it as a beam with a preferred curvature φ and persistence length L_{p}. As described in Fig. 2 *D*, the PF is straight and bound by lateral interactions with neighboring PFs for $z < 0$. For $z > 0$, we assume that there are no interactions to neighboring PFs so that the PF can bend freely. The PF conformation is described by the local bending angle $\theta(s)$ at the position s along the PF. The PF curvature is obtained by the derivative $\dot{\theta}(s) = \mathrm{d}\theta/\mathrm{d}s$. In the absence of an external force, the PF has a preferred (or spontaneous) constant curvature φ. Let the Ndc80 complex attach at the position $s = s_0$ and apply a force F in the z direction. Then, the total energy is the sum of bending and stretching energies,

$$E_{\mathrm{PF}} = \int_0^L \left(\frac{\alpha}{2}(\dot{\theta} - \varphi)^2 - zF\delta(s - s_0)\right)\mathrm{d}s, \quad (13)$$

with the bending stiffness $\alpha = k_{\mathrm{B}}TL_{\mathrm{p}}$ and the total length L of the free PF; the δ function describes a point force applied at $s = s_0$. The PF is dominated by the interplay of bending and stretching energy such that we can neglect thermal fluctuations and obtain the PF stiffness from the configuration minimizing the total energy E_{PF}. Since the force F only affects the shape of the PF for $s < s_0$ while the curvature at $s > s_0$ stays φ, we have to minimize the PF energy with boundary conditions $\theta(0) = 0$ and $\dot{\theta}(s_0) = \varphi$. The energy minimizing PF configuration satisfies the Euler-Lagrange equation $\ddot{\theta} = \frac{F}{\alpha}\sin\theta$, which we solve numerically with a shooting method in order to fulfill the boundary conditions. The effective deflection is given by the position of the point of force application on the z axis $z_0 = z(s_0)$. Finally, the effective stretching stiffness of the PF can be determined from the derivative of the deflection:

$$z_0(F) = \int_0^{s_0}\cos\theta(s)\,\mathrm{d}s,\ c_{\mathrm{PF}} = \left(\frac{\mathrm{d}z_0}{\mathrm{d}F}\right)^{-1}. \quad (14)$$

In the limit of strong forces ($F \gg \alpha/s_0^2 \sim 0.3-2$ pN), the PF is stretched, i.e., $\theta(s) \ll 1$, and we can approximate the differential equation by $\ddot{\theta} = (F/\alpha)\,\theta$, which can be solved analytically:

$$z_0(F) \approx s_0\left(1 - \frac{\varphi^2}{4s_0}\left(\frac{\alpha}{F}\right)^{3/2}\right),\ c_{\mathrm{PF}}(F) \approx \frac{8F^{5/2}}{3\varphi^2 a^{3/2}} \propto F^{\frac{5}{2}}. \quad (15)$$

We note the pronounced strain stiffening with $c_{PF}(F) \propto F^{5/2}$, which is a consequence of bending rigidity together with spontaneous curvature. The model depends on three parameters: the persistence length L_p, the preferred curvature φ, and the point of force application s_0. Following the results of in vitro experiments (34), we use $L_p = 200$ nm and $\varphi = 20^\circ$ per dimer throughout the paper. We assume that the Ndc80 complex can attach to the curved free part of the PF at a point s_0 that has to be smaller than the length L of the curved part of the PF. This assumption will be revisited in the "discussion" section. McIntosh et al. (34) measured lengths L in the range of 10–80 nm for depolymerizing MTs in vitro. In this paper, we will use estimates $s_0 = 20$ nm and $s_0 = 50$ nm.

PEG stiffness

We estimate the PEG stiffness on the basis of the extended FJC model for the elasticity of PEG provided by Oesterhelt et al. (35). Since PEG that is dissolved in water undergoes a conformational transition from a helical to a planar structure when tension is applied, the model includes two distinct bond lengths, one for each of the conformations. Assuming a Boltzmann distributed ratio of helical and planar monomers, the authors obtain an effective bond length that depends on the applied force. We derive the PEG stiffness c_{PEG} from the extension-force relation $L(F)$ that is given in Eq. 2 in (35):

$$c_{PEG}(F) = \left(\frac{dL(F)}{dF}\right)^{-1}. \tag{16}$$

For the PEG-2000 used in our experiments, we evaluate the equations with a number of $N_S = 45$ segments while keeping the other parameters as listed in (35) (see Supporting material for more details).

RESULTS

Strain stiffening can be observed for several Ndc80 variants

The ability of different Ndc80 variants to track and stall depolymerizing MTs has been examined in two previous studies (7,10). Here, we re-examined the underlying data to determine effective stiffnesses for wild-type Ndc80 complexes (Ndc80^{wt}) as well as three variants, including Ndc80 complexes with a phosphorylated tail (Ndc80^{P}), Ndc80 complexes, whose tail has been truncated (Ndc80$^{\Delta 80}$), and Ndc80 complexes with mutated CH domains and a greatly reduced MT binding (Ndc80^{CHmut}).

In (7), the stiffnesses for the wild-type Ndc80 were determined from the variance of bead positions during MT stall, which gave one stiffness per experiment. These experiments revealed stiffening under force. However, the stall force is related to the Ndc80 density on the bead and therefore probably also to the number of Ndc80 complexes that are attached to the MT. For this reason, we can not determine whether the stiffening is an intrinsic property of the Ndc80 complex and/or the PF, or whether it arises from a higher number of parallelly attached Ndc80 complexes.

To settle this issue, we need to time trace the stiffness throughout a single experiment. We achieve this by binning the data of bead positions in intervals of 0.1 s and determining an effective stiffness and applied force for each interval separately using the procedure described in the "materials and methods" section. This allows us to determine the stiffness while the depolymerizing MT pulls the attached bead and thereby increases the force. In other words, we measure the stiffness for different forces in a single experiment and, thus, with a fixed Ndc80 density on the bead. The results of this analysis are shown in Fig. 3 *A–D* for four representative examples, as well as in Fig. S1 in the Supporting material for 12 further examples.

For each of the Ndc80 variants, there are experiments in which we detect a smooth stiffening behavior when the tension increases, but there are also experiments that do not exhibit any stiffening at all, as in Fig. 3 *D*. Based on previously observed absence of stiffening during lateral bead-MT attachment (7), we interpreted experiments without stiffening as laterally attached and excluded them from the following analyses. We note that our main results and conclusions that we derive in the following are unchanged if the experiments without stiffening are included; see Fig. S2 in the Supporting material. Experiments with observed stiffening were interpreted as resulting from the end-on attachment that is sketched in Fig. 2 *A* and analyzed further. Stiffening was observed in 32 of 48 experiments with Ndc80^{wt}, eight of 24 with Ndc80$^{\Delta 80}$, 12 of 32 with Ndc80^{CHmut}, and 14 of 29 with Ndc80^{P} (see Fig. 3 *E*). Calibrations of QPD voltage to obtain bead displacement, as well as calibrations of trap stiffness as a function of bead's displacement from the trap center, were linear within 200 nm from the trap center. Therefore, displacements of a bead from the trap center that exceeded 200 nm were excluded from the following analyses of the strain stiffening.

In the experiments that exhibit stiffening, the MT depolymerization typically stalls at some force for Ndc80^{wt} and Ndc80^{P}, but not for Ndc80$^{\Delta 80}$ and Ndc80^{CHmut}, where the bead often detaches before it can stall the MT (see Fig. 3 *B*), and the stalls that occur are shorter (10). This indicates that the detachment force for Ndc80$^{\Delta 80}$ and Ndc80^{CHmut} is usually below the MT stall force under the experimental conditions applied. Therefore, a systematic analysis of these mutants is only enabled by time tracing of the stiffness prior to detachment, as measurements in a stalled state are rarely possible. The decrease in detachment force also confirms that the combination of the tail and the CH domain is essential for force-resisting attachment (10).

A stall can be interrupted in two possible ways: either the bead detaches from the MT (Fig. 3 *A*) or MT growth is rescued (Fig. 3 *C*). In the first case, the bead relaxes immediately to the equilibrium position of the optical trap and the measured stiffness is the trap stiffness c_{trap}. In case of a rescue, the bead stays attached to the polymerizing MT and the tension decreases until the MT undergoes a catastrophe and starts depolymerizing again. This will allow us to investigate stiffening of the Ndc80 complexes in dependence of the dynamic state of the MT in the following

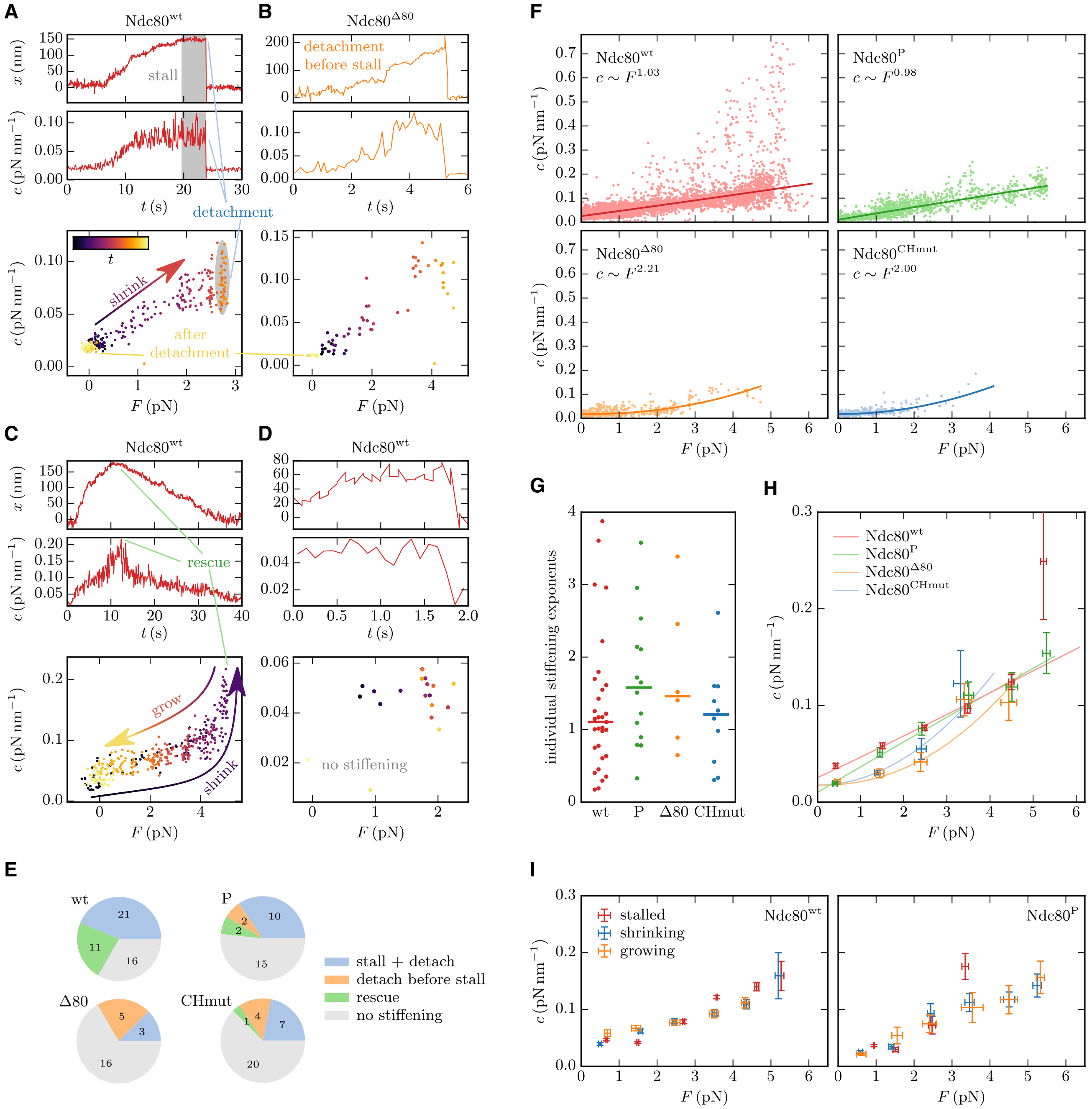

FIGURE 3 (*A–D*) Four representatives for typical behavior during single experiments. Further examples, including Ndc80^{CHmut} and Ndc80^{P}, are shown in Fig. S1 in the Supporting Material. For each experiment, the upper two plots show the piecewise linearized bead position and the stiffness over time, while the stiffness-force relation is depicted in the bottom plot, in which the time is represented by the color gradient. When the force is increased due to the shrinking MT, we can observe stiffening in most experiments (*A–C*). Shrinking usually is followed by a stall phase with constant force and constant stiffness (gray), except for experiments with Ndc80$^{\Delta 80}$ (*B*) and Ndc80^{CHmut}, in which the bead often detaches before reaching the stall force. The stall is terminated either by detachment (*A*) or by MT rescue (*C*). After detachment the bead relaxes to $x = 0$ and we measure the trap stiffness. When the MT is rescued and grows, the stiffness-force relation traces the same curve as during depolymerization. (*E*) Observed frequencies of the typical events described in (*A*)–(*D*). (*F*) Robust power fits. For each Ndc80 variant, the combined stiffness data from all experiments (dots) are robustly fitted with power functions $c(F) = aF^m + c_0$ (lines) as described in the Supporting Material. The offset c_0 should respect the trap stiffness. (*G*) Stiffening exponents m that result from applying the power fits to those single experiments, where a proper stiffening is recognizable and the bead was attached to the MT for at least 1 s, so that there are $\geq$ 10 stiffnesses to fit to. The horizontal bars mark the medians. (*H*) Summarized stiffness-force relations. The error bars represent the standard deviation of the mean in F and in c of the points within each bin, where each experiment is considered as an independent event. The lines show the power fits from (*F*). (*I*) Stiffnesses separately evaluated for stalled, shrinking, and growing MTs. Error bars as described in (*H*). Ndc80$^{\Delta 80}$ and Ndc80^{CHmut} are not included here because of rare occurrence of rescues. To see this figure in color, go online.

section. The four typical behaviors (stiffening during stall followed by detachment or rescue, detachment before stall, or no stiffening) that are described above and in Fig. 3 *A–D* can be observed for each of the Ndc80 variants with frequencies as summarized in Fig. 3 *E*.

Another advantage of our time-tracing method is that we generate many more data points from the same number of experiments. While, for example, 53 stiffnesses of $Ndc80^{wt}$ were determined in (7) from stalling MTs, time tracing generates 4403 stiffnesses from the same experiments (see Fig. S3 in the Supporting material). The cumulated force-stiffness data, which are collectively depicted in Fig. 3 *F* for each Ndc80 variant, reveal the strain stiffening more clearly than the sole use of the stalls in Ref. 7 and allow for a more thorough analysis and interpretation.

In order to further characterize strain stiffening, we fit power law functions $c(F) = aF^m + c_0$ to the collective data by use of a robust regression minimizing the Huber loss (36) (see Fig. 3 *F* and Supporting material). The exponent m characterizes the observed strain stiffening behavior. We account for the trap stiffness by the offset c_0 for which the fits yield results that, indeed, lie in a range 0.01–0.025 pN nm^{-1}, which is in accordance with the trap stiffnesses determined during calibration. While the stiffnesses exhibit a linear dependence on force with exponents m around unity for $Ndc80^{wt}$ and $Ndc80^{P}$, the stiffness–force relations of $Ndc80^{\Delta 80}$ and $Ndc80^{CHmut}$ have a roughly parabolic shape ($m \approx 2$). We note that, for $Ndc80^{\Delta 80}$ and $Ndc80^{CHmut}$, there are few data for high forces, making the stiffening exponents less reliable.

The stiffening exponents determined in Fig. 3 *F* are the result of a fit to the combined data from several experiments and may be biased by the correlation between the stall force and the Ndc80 densities on the bead. Our new time-tracing analysis, however, dampens this bias compared with the sole analysis of the stalls as stiffnesses are determined for small forces but at high Ndc80 densities during MT depolymerization in the respective experiments. We also apply the power law regressions to the stiffness-force relations of single experiments. Then, we find most stiffening exponents in a range between 0.5 and 2 but also detect outliers below this range and close to 4 (Fig. 3 *G*). Finally, we evaluated the stiffness-force relations separately for different populations of beads. Since beads within the same population were prepared under the same conditions, they can be assumed to be coated with a similar number of Ndc80 complexes. Although the populations had very different mean Ndc80 densities, the separate evaluation does not reveal a correlation between Ndc80 density and the stiffening behavior, as shown by Fig. S4 in the Supporting material. Therefore, we exclude that the linear stiffening is a result of the correlation between stall force and number of Ndc80 complexes per bead.

For a better visibility and comparability of the strain stiffening of the different Ndc80 variants, we summarize the stiffness measurements in Fig. 3 *H* by binning the time-traced stiffnesses of all experiments in bins of 1 pN width and averaging force and stiffness in each of these bins. We find that all variant Ndc80 complexes exhibit similar strain stiffening. Both truncating the tail or modifying the CH domain slightly decrease the stiffness compared with the wild type, especially for small forces. This indicates that, in all four Ndc80 variants, the remaining intact common parts play a central role in strain stiffening. Differences between variants are due to changes in the Ndc80-MT bond and will be addressed in the "discussion" section.

Measured stiffness is independent of MT state

When an MT is rescued after the stall, as in the experiment depicted in Fig. 3 *C*, the time-traced stiffness follows the same stiffness-force relation in the shrinking and growing states before and after rescue, respectively. The same observation can be made in experiments with several consecutive rescues and catastrophes. This indicates that the stiffness is independent of the MT state; i.e., on whether the Ndc80 complexes are attached to a shrinking, stalled, or growing MT. To address this question systematically, we identify the phases of shrinking, stalled, and growing MTs in the single experiments, and bin the stiffnesses analogously to Fig. 3 *H* but separately for each of the three MT states. The results in Fig. 3 *I* do not show a significant dependence on the MT state for any of the four Ndc80 variants. The independence of Ndc80-MT linker stiffness from MT state indicates that PFs either do not contribute to the total stiffness (because $c_{PF} \gg c_{tot}$), or that, if they do contribute, their mechanical properties do not depend on whether the MT is growing or shrinking. The stiffness contributions of PFs are investigated below in more detail.

PLL-PEG is too stiff to contribute to the measured stiffnesses

In the following sections, we attempt to explain the observed strain stiffening by modeling the elastic elements between the bead and the MT that add up to the stiffness $\tilde{c}(F)$ as shown in Fig. 2 *B* and Eq. 2. We start with the PLL-PEG linkage to the bead, whose contribution to the measured stiffnesses should be clarified in addition to those from the Ndc80 complex or the PF. PLL-PEG consists of a poly-l-lysine (PLL) backbone and polyethylene glycol (PEG) side chains. While the PLL backbone is adsorbed on the glass bead in the experiment, the flexible PEG chains can bind the Ndc80 oligomers.

From force-extension curves measured in AFM experiments (37), stiffness of PLL adsorbed on a flat Nb_2O_5 surface can be estimated as ~100 pN nm^{-1}, which is three orders of magnitude above the stiffnesses observed in our experiments. Oesterhelt et al. (35) successfully described the elasticity of single PEG polymers by means of an

extended FJC model with a bond length that follows from a two-level system. Applying this model to the PEG-2000 with $N_S = 45$ segments used in our experiments, we obtain a force-free stiffness of 1.38 pN nm^{-1} that increases under tension, as shown in the Supporting material.

We conclude that both the PLL backbone and the PEG chains are too stiff to make a significant contribution to the observed strain stiffening and can be omitted in the following analysis ($c_{PEG}(F) \gg \tilde{c}(F)$ in Eq. 2.

Stiffening is a direct consequence of the Ndc80 structure

After eliminating a possible contribution of the PLL-PEG, we will next include the remaining elements (Ndc80 complex, MT, PF) step by step to discuss their relevance for the observed strain stiffening. We start with the Ndc80 complex, which is the most promising and interesting candidate because of its flexible structure and its force transmitting role in the mitotic spindle.

We model the Ndc80 complex as an FJC with two flexibly connected stiff arms (see Fig. 2 *C*), resulting in a stiffness $c_{Ndc}(F)$ as given by Eq. 11. We have to take into account that there might be n Ndc80 complexes attached in parallel. For this situation, we assume that each Ndc80 complex has the same elongation so that the force F is shared equally by the attached complexes and the total stiffness is given by $n\, c_{Ndc}(F/n)$ (see Eq. 3). In Fig. 4 *A*, we compare the total stiffness of n parallel Ndc80 complexes with the measured stiffnesses of Ndc80^{wt}. For any n, the model results are about a factor of 2–4 larger than the measured values. Moreover, the two-segment Ndc80 model results in a strain stiffening exponent $m = 2$ in Eq. 12, which is larger than the measured values $m \approx 1$ in Fig. 3 *E*. We conclude that the stiffness of the Ndc80 complex alone can explain strain stiffening but is not sufficient to explain the measured stiffnesses quantitatively.

We note that the experimental values in Fig. 4 still contain the trap stiffness, which is not included in the theoretical curves. We refrained from correcting the experimental values for the trap stiffness, as trap stiffness and time-traced stiffnesses were determined by different methods and are, therefore, difficult to compare: while the time-traced data were obtained from the equipartition theorem, as described in the "materials and methods" section, the trap stiffness, which was also used for force determination, was calibrated from the power spectral density (PSD) (38–40). In conclusion, one should keep in mind that the wild-type stiffness values in Fig. 4 probably need to be shifted slightly downward by an amount of approximately 0.02 pN nm^{-1}.

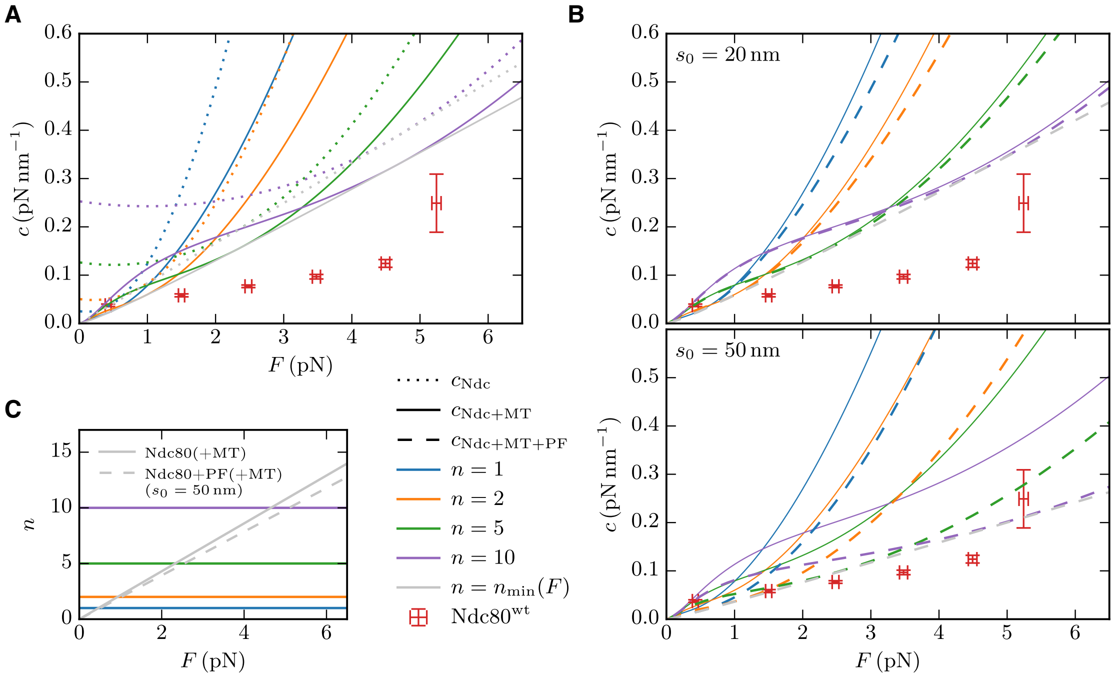

FIGURE 4 (*A*) The stiffness of n parallel Ndc80 complexes $n\, c_{Ndc}(F/n)$ according to Eq. 10 (dotted lines) is by a factor of 2–4 larger than the measurements with wild-type Ndc80 (red, see Fig. 3 *H*). Adding the MT stiffness in series (solid lines) reduces the stiffness, but not sufficiently to match the measurements. The theoretical stiffness is minimized when the number of attached Ndc80 complexes increases linearly with the force ($n_{min}(F)$, gray line). (*B*) Stiffness of an MT in series with n parallel Ndc80 complexes, each of which is in series with a PF (dashed lines). Top: when the attachment on the curved PF has a distance of $s_0 = 20$ nm from the straight MT lattice, the stiffness only differs slightly from the stiffness of an MT in series with just the Ndc80 complexes (solid lines). Bottom: for $s_0 = 50$ nm, the PF does have a significant contribution to the total stiffness. However, the combined stiffnesses of Ndc80 complexes and PFs are still larger than the experimental outcomes for wild-type Ndc80. The Ndc80^{wt} values in (*A*) and (*B*) are not corrected by the trap stiffness (see text). (*C*) Force-dependent number of attached Ndc80 complexes that minimizes the theoretical stiffness ($n_{min}(F)$, gray). To see this figure in color, go online.

The fact that the Ndc80 model gives larger stiffnesses than measured motivates the consideration of the additional elastic elements from the MT, the PFs, and the Ndc80-MT bonds in the total stiffness in Eq. 4. These elements are in series to the Ndc80 complex and potentially reduce the total stiffness of the entire Ndc80-MT link.

Entropic MT stiffness

Next, we consider the contribution of the MT, which can be attached to several Ndc80 complexes. While a purely mechanical MT stiffness that follows from stretching or bending the MT can be ruled out to contribute significantly (see Supporting material), the MT also has an entropic elasticity from thermal fluctuations. To estimate the entropic stiffness, we describe the MT as a worm-like chain by means of the force-extension relation in Eq. 5 taking into account a length-dependent persistence length (29). The relevant MT length is the free part of the MT; i.e., the contour length between the fixed MT seed and the MT tip. As a further consequence of the length-dependent persistence length, the resulting MT stiffness in Eq. 6 becomes length independent for small MT lengths $L \ll L_0 = 21$ μm. Therefore, the MT stiffness can be assumed to be the same in all experiments, where typical MT lengths are between 3 and 10 μm, and we use $L = 10$ μm in the following as a general representative.

The entropic stiffness of the MT is of the same order of magnitude as the Ndc80 stiffness, which is somewhat surprising for an MT that is by a factor 100 shorter than its persistence length. Consequently, the stiffness of n parallel Ndc80 complexes alone is significantly reduced when the MT stiffness is added in series (see Fig. 4 *A*). Interestingly, the combination of a single MT with stiffening exponent 3/2 and several ($n \gtrsim 5$) Ndc80 complexes stiffening with F^2 results in a roughly linear behavior in the examined force range, which might explain the measured stiffening exponents in Fig. 3 *E*. Despite the overall stiffness reduction, our model predictions are still too large compared with the measurements when only the MT and the Ndc80 complexes are considered. Therefore, we speculate in the next step that the Ndc80 complexes bind to the curved parts of the PFs resulting in an effective stiffness from straightening the PFs.

Only long protofilaments are flexible enough to reduce the total stiffness

We assume that forces recorded in our experiments are produced by tubulin protofilaments bending outwards from the MT lattice when an MT shortens. We always record stall events after a force has been developed; i.e., we do not observe bead-bound Ndc80 preventing MT shortening without force development. Therefore, we hypothesize that bead-bound Ndc80 interacts with bent protofilaments during stall. We also assume that, during stall, the MT-generated force is equalized by the returning force of the optical trap directed along the MT axis in the opposite direction.

Since the PFs are bent out of their preferred curvature, a restoring force on the Ndc80 complexes builds up, governed by an effective stiffness c_{PF} for strains in the axial direction. In the "materials and methods" section, we quantified this stiffness with a simple beam model (see Fig. 2 *D*) in Eq. 14. This model depends on three parameters: the preferred curvature φ of the PF, its persistence length L_{p} that defines the bending stiffness $\alpha = k_{\mathrm{B}}TL_{\mathrm{p}}$, and the position s_0 along the free part of the PF where the Ndc80 complex attaches (i.e., where the force is applied). While the first two parameters as well as the length of the free curved parts of the PF are well described for MTs in vitro (34), a fixed attachment position s_0 of Ndc80 complex to the PF is not known, and we use typical values $s_0 = 20$ nm and $s_0 = 50$ nm.

In Fig. 4 *B*, we plotted the stiffnesses of n parallel Ndc80 complexes with c_{Ndc} that are each in series with a PF c_{PF}. We see that the influence of PF bending is negligible for $s_0 = 20$ nm, whereas for $s_0 = 50$ nm the PF significantly reduces the total stiffness close to the measured wild-type values. We conclude that the PF stiffness is only relevant when the Ndc80 complex is attached near the end of a sufficiently long PF. An upper bound for s_0 is given by the total length of the curved part of the PF for which values of $L = $ 10-80 nm have been measured in vitro (34).

Catch-bond behavior lowers the stiffness of parallel Ndc80 complexes

So far, we have assumed a *fixed* number n of parallel Ndc80 complexes over the whole range of applied forces. We now analyze how our model behaves when the number n of attached Ndc80 complexes depends on the applied force, $n = n(F)$. In principle, both a catch bond and a slip bond mechanism are conceivable for the Ndc80-MT bond. A catch (slip) bond is characterized by a binding affinity that increases (decreases) under tension (41). In our model, a catch (slip) bond implies a monotonically increasing (decreasing) force-dependent number of attached Ndc80 complexes $n(F)$.

We find that a force-dependent $n(F)$ can lower the effective total stiffness of the entire Ndc80-MT link. The minimal cooperative stiffness that can be realized is the envelope of the stiffness-force relations $c_n(F)$ for different n in Fig. 4 *A* and *B*. The envelope of the $c_n(F)$ relations is obtained by a force-dependent number $n_{\min}(F)$ of attached Ndc80 complexes that minimizes the stiffness $c_n(F)$ for each F. The resulting $n_{\min}(F)$ is shown in Fig. 4 *C* and increases linearly, which also implies a catch-bond behavior. For long PFs ($s_0 = 50$ nm), the envelope stiffness $c_{n_{\min}}(F)$ has actually the correct order of magnitude compared with the experimental data shown in Fig. 4 *B*. The number $n_{\min}(F)$ also remains below $n = 15$ in the examined force range, which is in agreement with estimates

for the number of Ndc80 complexes in the proximity of the MT given the expected Ndc80 density on the bead (5,7).

Apart from the absolute stiffness values, a model should reproduce the observed roughly linear strain stiffening behavior with the exponent m around unity (see Fig. 3 *E*). Our theory, on the other hand, predicts exponents of 3/2, 2, and 5/2 for the MT, the Ndc80 complexes and the PFs, respectively. The relation $n(F)$ can change the latter two stiffening exponents. From Eq. 3, one can see that a linearly increasing $n(F)$ actually implies a linear stiffening as observed in our experiments since $F/n(F) = \text{const}$ and, therefore, $\tilde{c}_n(F) = n(F)\tilde{c}(\text{const}) \propto n(F)$.

For a slip bond, on the other hand, where $\partial_F n(F) < 0$ and $n(F) \rightarrow 0$ for large forces by definition, the argument in Eq. 3, $F/n(F)$, increases rapidly. As a consequence, we can approximate c_{Ndc} and c_{PF} with the power laws from Eqs. 12 and 15, respectively, so that

$$c_n(F) \sim n(F)\left(\frac{F}{n(F)}\right)^m = \frac{F^m}{(n(F))^{m-1}} > \mathcal{O}(F^m), \tag{17}$$

We conclude that any kind of slip bond will increase the stiffening exponent for large forces. Stiffening exponents below the values $m = 2$ for Ndc80 stiffness or $m = 2.5$ for PF stiffness, as obtained in the experiments, are indicative of a catch-bond mechanism. For the time traces from single experiments in Fig. 3 *E*, 79 % of the stiffening exponents lie below 2.

DISCUSSION

The novel analysis of time traces of bead positions in optical trapping experiments allows us to time trace the stiffness during single experiments and go beyond an analysis limited to the MT stall state (7). This enabled us to increase the available number of stiffness measurements by one to two orders of magnitude and study strain stiffening of wild-type Ndc80 in more detail. It also enabled us to study strain stiffening of Ndc80 variants with a truncated tail or with a mutated CH domain, both of which typically detach before the MT has been stalled, and to explore strain stiffening selectively in the shrinking, stalled, and growing state of MTs.

We found that all mutant Ndc80 complexes exhibit strain stiffening hinting at a central role of the Ndc80-Nuf2 coiled coil, rather than the MT binding sites in the CH domain and the N-terminal tail. The measured stiffnesses of the entire Ndc80-MT link are lower than theoretical predictions from our Ndc80 model. Therefore, additional elastic elements in the Ndc80-MT link or a force-dependent number of attached Ndc80 complexes should be relevant. We could exclude the PLL-PEG connection between Ndc80 complexes and the bead, which is too stiff to contribute. Effects from the MT and from protofilament flexibility are necessary for the model to conform to experimentally measured stiffnesses, and the observed linear stiffening may be explained by an increasing number of attached Ndc80 complexes under force (catch bond). Moreover, stiffness differences between variants allow us to draw some conclusions on the Ndc80-MT bond.

Effects from protofilaments

Time tracing of the stiffness allowed us to study strain stiffening selectively in the shrinking, stalled and growing state of MTs. We found no significant stiffness differences between MT states (see Fig. 3 *C* and *H*), leading to the conclusion that the stiffness of the bent PFs is either too large to contribute significantly to the total stiffness or that it does not depend on the MT's polymerization state.

Our PF model showed that the PF contribution to the overall stiffness depends strongly on the length of the PFs and the position along the PF where the Ndc80 complex is attached. An attachment point s_0 close to the straight part of the PF ($s_0 \leq 20$ nm) predicts a negligible contribution of the PF. It has been shown that single Ndc80 complexes bind to straight PFs but show a much weaker affinity for curved PFs (6,31). The results of (31) also suggest that the weaker affinity is due to a missing interaction of the CH domain with a curved PF so that a weaker bond is formed by the N-terminal tail alone. This is supported by the results in Fig. 3 *E*, which show that Ndc80$^{\Delta 80}$ with truncated tail enters a state of stall, which requires binding to the MT, less frequently. There are, however, differences. Our stiffness measures derive from experiments where Ndc80 multimers were used that were able to tip-track, in contrast to the single Ndc80 complexes in (31) and (6). Moreover, these experiments were not performed with full wild-type Ndc80 complexes but with Ndc80^{bonsai}, lacking almost the entire coiled coil region (31), or with Ndc80^{broccoli}, lacking Spc24 and Spc25 (6). Together, these differences leave room for speculating that our experimental conditions allow for Ndc80 binding to curved PFs. For instance, the Ndc80 complexes may bind initially to a straight PF, which becomes curved at the binding site during depolymerization while the Ndc80 complex stays attached.

Attachment of Ndc80 to long flaring PFs with attachment lengths around $s_0 = 50$ nm are predicted to reduce the stiffness of the Ndc80-MT link to values close to the measured stiffnesses (Fig. 4 *B*). This is consistent with the conclusion that PFs contribute to the total stiffness but have identical elastic properties during MT shrinkage, stall, and growth, and is also in agreement with the recent observation that the curvature of PFs is the same during polymerization and depolymerization (34).

Number of attached Ndc80 complexes and potential catch-bond mechanism

The number n of simultaneously attached Ndc80 complexes could not be directly inferred from the experiments in (7)

and (10). From the known Ndc80 densities on the bead, we can estimate that one to four Ndc80 trimers (i.e., three to 12 Ndc80 complexes) are in the vicinity of the MT end and can potentially bind (7).

From the equal stiffness-force relations before and after a rescue, we also conclude that the number of attached Ndc80 complexes is the same during polymerization and depolymerization. Dynamically, it is possible that the number of attached Ndc80 complexes changes in a force-dependent manner between MT catastrophes and rescues. For force-free detachment and attachment of a single Ndc80^{wt} complex, timescales of $\tau_{\text{off}} = 1.6$ s and $\tau_{\text{on}} = 0.4$ s, respectively, have been found (7). With a phosphorylated tail, τ_{off} is supposed to be smaller (12). Since the durations of MT depolymerization vary in a wide range from less than 1 s up to ~ 100 s, both a constant and a dynamic number of attached complexes are possible.

Our modeling results showed that the absolute stiffness values as well as the roughly linear strain stiffening relation (see Fig. 3 *E*) are best reproduced by a force-dependent number $n(F)$, which increases linearly with force (see Fig. 4 *B*) implying a catch-bond mechanism. Among the stiffening exponents for the time traces from single experiments in Fig. 3 *F*, 79% of the strain stiffening exponents lie below 2, which is indicative of a catch-bond-like behavior. As already noted above, the linear stiffening may also be an apparent phenomenon that results in the examined force range from the combination of the different stiffening behaviors of a single MT and a sufficiently large constant number of Ndc80 complexes and PFs.

Assuming that Ndc80 forms a catch bond to the MT, the question arises how this mechanism could work. The whole kinetochore, consisting of several additional proteins, has been proposed to act like a catch bond (42). It is widely assumed that the Aurora B kinase, which was not present in our experiments, is important in the kinetochore's catch-bond mechanism (43–45). Kinetochores purified from *Saccharomyces cerevisiae*, however, were shown to build catch bonds with MTs in vitro even without Aurora B activity (46). Importantly, the catch-bond behavior of the purified yeast kinetochores was abolished when Dam1 or Stu2 were absent, even in presence of Ndc80 (46,47). There is evidence that Ndc80 stretching correlates with MT binding as the Ndc80 complex may exist in an auto-inhibited bent conformation with reduced MT binding capacity (48) and that the Ndc80 complex bends ("jackknifes") upon detachment (49). This suggests that the binding affinity for MTs increases when the Ndc80 complex is stretched, which would further imply that Ndc80 has an intrinsic catch-bond-like mechanism. To what extent this effect is relevant for our experiments is unclear, as, according to our FJC model, the Ndc80 complex is already stretched by small forces. For instance, at $F = 1$ pN we find a mean angle of $(44 \pm 23)^\circ$ between the arms $\vec{a}$ and $\vec{b}$ (where the angle is 0° for a maximally stretched Ndc80 complex). Speculating on other possible catch-bond mechanisms, it is conceivable that by stretching the unstructured tail additional binding sites become available which are concealed in the entangled tail at low forces. Force-enhanced adhesion by unfolding is a common catch-bond mechanism (50); examples are the von Willebrand factor (51,52) and α-catenin bonding to F-actin (53) in the cytoskeleton. In conclusion, an intrinsic catch-bond mechanism of Ndc80 complexes is compatible with current knowledge and worth considering as one of the ingredients that define the characteristics of Ndc80 strain stiffening. Such a mechanism could also contribute to the catch-bond behavior of the entire kinetochore.

We assumed throughout the paper that the applied force is shared equally among the Ndc80 complexes attached in parallel so that each complex and each PF has the same elongation. If this assumption is lifted, the system will be dominated by a few very stiff Ndc80 complexes, which are the ones with the largest stretch. It is possible that the linker extensions approach a uniform distribution under force; for instance, because some extremely stretched Ndc80 complexes detach from the shrinking MT. Then, despite detachment of a few linkers, the number of linkers with a relevant stretch increases under force. In conclusion, such a mechanism would result in an apparent catch-bond behavior without the need for individual Ndc80-MT catch bonds.

Structure of Ndc80-MT bond and role of the N-terminal tail in MT binding

Stiffness differences between the wild-type Ndc80^{wt} and mutants Ndc80$^{\Delta 80}$ and Ndc80^{CHmut} reflect roles in MT binding of the N-terminal tail and the CH domain, respectively. These differences will allow us to draw some conclusions on the Ndc80-MT bond, whose exact mechanism is still unknown. The CH domain binds between two tubulin monomers via a structural element named the "toe" (31). The N-terminal tail also supports MT binding, but its exact role is still under debate. Three models are currently discussed (23,54): direct binding to the MT lattice, cooperativity and clustering by interactions with neighboring Ndc80 complexes, and co-factor recruitment. Since no co-factors were present in our experiments, we will concentrate on the former two models.

Our comparative study of Ndc80^{wt}, Ndc80$^{\Delta 80}$, and Ndc80^{CHmut} allows us to dissect the role of the tail and the CH domain on Ndc80-MT binding. Both Ndc80 mutants displayed decreased stiffnesses compared with the wild type (Fig. 3 *G*), an effect we ascribed either to a softened Ndc80-MT bond or to a reduced number of attached Ndc80 complexes. In the elastic toy model in Fig. 5 *A*, both the CH domain and the tail of Ndc80^{wt} bind to the PF, so that they can be represented by two parallel springs that add up to the total bond stiffness c_{bond}. Due to the parallel

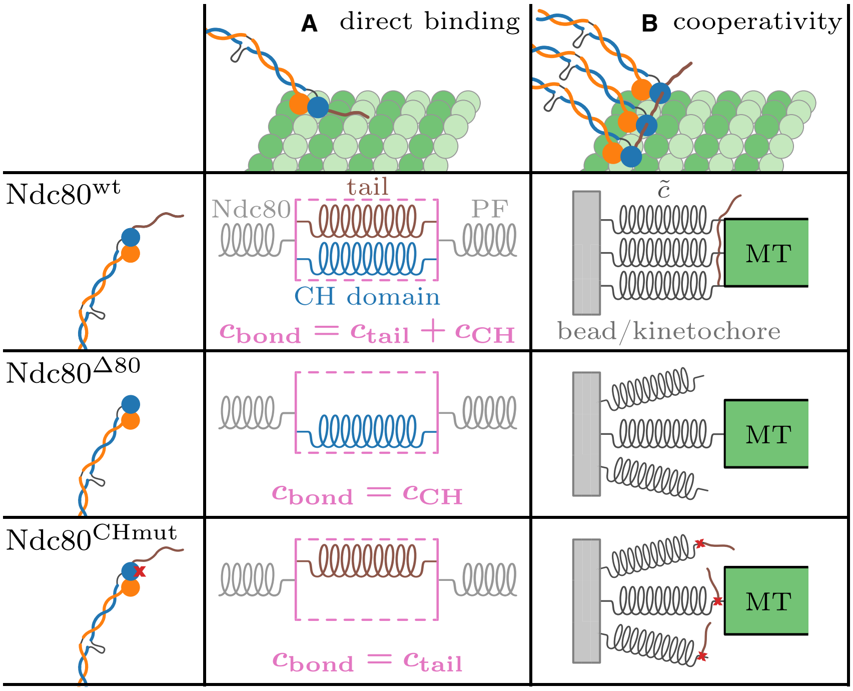

FIGURE 5 Models of the Ndc80-MT bond for potential roles of the N-terminal tail. (*A*) When the tail binds directly to the PF, an elastic model of the Ndc80-MT bond comprises the tail and the CH domain as two parallel springs. If the tail is deleted ($Ndc80^{\Delta 80}$) or the CH binding domain is blocked ($Ndc80^{CHmut}$), either of those springs is missing, which lowers the bond stiffness c_{bond} and thereby the total stiffness $\tilde{c}$. (*B*) In the case of an interaction of the tail with CH domains of neighboring Ndc80 complexes, the number of parallelly attached linkers is reduced for the two mutants $Ndc80^{\Delta 80}$ and $Ndc80^{CHmut}$. To see this figure in color, go online.

arrangement, lack of either of the two springs in $Ndc80^{\Delta 80}$ or $Ndc80^{CHmut}$ reduces the bond stiffness, predicting overall stiffnesses as depicted in Fig. 3 *G*. If both the CH domain and the N-terminal tail bind directly to the MT lattice, the two Ndc80 mutants probably have a reduced MT binding affinity. Therefore, in the direct binding model, the reduction of the overall stiffness could be a consequence of a reduced number of attached Ndc80 complexes as well as of a reduction of the individual bond stiffnesses.

When the N-terminal tail interacts with neighboring Ndc80 complexes instead of the tubulin, the induced cooperativity supports parallel attachments of multiple $Ndc80^{wt}$ complexes, see Fig. 5 *B*. Deletion of the tail or mutation of the CH domain may impair the cooperative behavior, with the result that fewer linkers connect the bead (or the kinetochore) with the MT and the overall stiffness decreases (for small and medium forces; see Fig. 4). While there is evidence supporting a contribution of the tail to cooperative MT binding, there is at present no clear evidence indicating that the Ndc80 CH domain contributes to binding cooperativity.

Tail phosphorylation was shown to reduce the MT binding affinity of the Ndc80 complex (2,11–14). Moreover, the durations of the MT stalls are shorter when the Ndc80 tails are phosphorylated (10). This suggests that phosphorylation impedes the function of the tail similarly as if the tail is truncated. However, in contrast to $Ndc80^{\Delta 80}$, the stiffness of $Ndc80^{P}$ is not significantly reduced compared with $Ndc80^{wt}$, see Fig. 3 *G*. It remains an open question how these observations are compatible with the binding models depicted in Fig. 5.

Comparison of model and experiment

Our model can explain the strain stiffening from the structure of the Ndc80 complex and the bending elasticity of long flaring PFs. If we allow for a force-dependent number of bound Ndc80 complexes, a linearly increasing number of bound complexes (requiring a catch-bond mechanism) also reproduces the observed roughly linear strain stiffening relation. Ndc80, by itself or in series with PFs, gives absolute stiffness values that are still above the measured stiffnesses. Including a force-dependent number of bound Ndc80 complexes gives values that are close but still slightly higher than measured. As already mentioned, the missing part might be the Ndc80-MT bond, which is additionally in series with the Ndc80 complex and the PF, as illustrated in Fig. 2. However, since the stiffness of the bond is hard to quantify due to the little knowledge of the exact binding mechanism, we did not go beyond the qualitative discussion in the previous section.

Regarding the Ndc80, we have also investigated possible effects from finite flexibility of the two Ndc80 arms by modeling both arms as semiflexible worm-like chains. We performed Monte Carlo simulations, in which each Ndc80 arm had the same persistence length L_p. Using persistence lengths in a realistic range of $L_p \gtrsim 100$ nm as they have been determined for other coiled coil proteins such as tropomyosin (55), our simulations lead to the conclusion that semiflexibility has only a minor effect on total stiffness (see Supporting material).

Finally, we need to discuss the accuracy of our experimental method. While the trap stiffness was calibrated by

fitting the power spectrum based on Brownian motion in a harmonic potential (38–40), this well-established method can not be applied for measurements of the anharmonic Ndc80 stiffness. Therefore, we had to determine the Ndc80 stiffness from the variance of bead position, which is a simpler and universal but less accurate approach. In particular, the measured overall variance contains a systematic noise that is added to the actual variance of bead position, which is why the stiffness tends to be underestimated by $k_B T/\mathrm{Var}(x)$ (56–58). This systematic underestimation, which we cannot quantify, might explain why our model tends to predict higher stiffnesses than experimentally measured.

CONCLUSIONS

In conclusion, we accumulated extensive stiffness data of the Ndc80-MT link by optical trapping methods in combination with a novel time-tracing analysis. We were able to study wild-type Ndc80 complexes and three variants. Our theoretical model for the Ndc80-MT link, which includes structure-based models of the Ndc80 complex, the MT, and flaring PFs, is able to explain the strain stiffening observed when Ndc80 is bound to shortening MT plus ends, and reproduces the correct order of magnitude of the stiffness. Thus, our results on the elastic properties further support these structural models from the mechanical point of view. Our model also reproduces the roughly linear strain stiffening behavior when taking a force-dependent binding affinity into account.

SUPPORTING MATERIAL

Supporting material can be found online at https://doi.org/10.1016/j.bpj.2022.09.039.

AUTHOR CONTRIBUTIONS

A.M., M.D., V.A.V., and P.J.H. conceived and designed the experiments. P.J.H. contributed reagents. V.A.V. performed and analyzed the experiments. J.K. and F.S. developed the theoretical model. F.S. analyzed the data and performed calculations. F.S. and J.K. wrote the original draft. A.M., V.A.V., and P.J.H. reviewed and edited the manuscript.

ACKNOWLEDGMENTS

V.A.V. and M.D. acknowledge support from European Research Council Synergy Grant MODELCELL (proposal 609822). A.M. acknowledges funding by European Research Council (ERC) through Synergy Grant 951439 (BIOMECANET).

DECLARATION OF INTERESTS

The authors declare no competing interests.

SUPPORTING CITATIONS

References (59–61) appear in the Supporting material.

REFERENCES

1. Musacchio, A., and A. Desai. 2017. A molecular view of kinetochore assembly and function. *Biology.* 6:5.
2. DeLuca, J. G., W. E. Gall, …, E. D. Salmon. 2006. Kinetochore microtubule dynamics and attachment stability are regulated by Hec1. *Cell.* 127:969–982.
3. Wei, R. R., P. K. Sorger, and S. C. Harrison. 2005. Molecular organization of the Ndc80 complex, an essential kinetochore component. *Proc. Natl. Acad. Sci. USA.* 102:5363–5367. https://doi.org/10.1073/pnas.0501168102.
4. Wang, H.-W., S. Long, …, E. Nogales. 2008. Architecture and flexibility of the yeast Ndc80 kinetochore complex. *J. Mol. Biol.* 383:894–903.
5. Powers, A. F., A. D. Franck, …, C. L. Asbury. 2009. The Ndc80 kinetochore complex forms load-bearing attachments to dynamic microtubule tips via biased diffusion. *Cell.* 136:865–875.
6. Schmidt, J. C., H. Arthanari, …, I. M. Cheeseman. 2012. The kinetochore-bound Ska1 complex tracks depolymerizing microtubules and binds to curved protofilaments. *Dev. Cell.* 23:968–980.
7. Volkov, V. A., P. J. Huis In 't Veld, …, A. Musacchio. 2018. Multivalency of NDC80 in the outer kinetochore is essential to track shortening microtubules and generate forces. *Elife.* 7:e36764.
8. Tien, J. F., N. T. Umbreit, …, T. N. Davis. 2010. Cooperation of the Dam1 and Ndc80 kinetochore complexes enhances microtubule coupling and is regulated by aurora B. *J. Cell Biol.* 189:713–723.
9. Lampert, F., P. Hornung, and S. Westermann. 2010. The Dam1 complex confers microtubule plus end–tracking activity to the Ndc80 kinetochore complex. *J. Cell Biol.* 189:641–649.
10. Huis In 't Veld, P. J., V. A. Volkov, …, M. Dogterom. 2019. Molecular determinants of the Saka-Ndc80 interaction and their influence on microtubule tracking and force-coupling. *Elife.* 8:e49539. https://elifesciences.org/articles/49539.
11. DeLuca, K. F., S. M. A. Lens, and J. G. DeLuca. 2011. Temporal changes in Hec1 phosphorylation control kinetochore-microtubule attachment stability during mitosis. *J. Cell Sci.* 124:622–634. https://doi.org/10.1242/jcs.072629.
12. Umbreit, N. T., D. R. Gestaut, …, T. N. Davis. 2012. The Ndc80 kinetochore complex directly modulates microtubule dynamics. *Proc. Natl. Acad. Sci. USA.* 109:16113–16118. https://doi.org/10.1073/pnas.1209615109.
13. Zaytsev, A. V., L. J. R. Sundin, …, J. G. DeLuca. 2014. Accurate phosphoregulation of kinetochore–microtubule affinity requires unconstrained molecular interactions. *J. Cell Biol.* 206:45–59.
14. DeLuca, K. F., A. Meppelink, …, J. G. DeLuca. 2018. Aurora A kinase phosphorylates Hec1 to regulate metaphase kinetochore–microtubule dynamics. *J. Cell Biol.* 217:163–177.
15. Hill, T. L. 1985. Theoretical problems related to the attachment of microtubules to kinetochores. *Proc. Natl. Acad. Sci. USA.* 82:4404–4408. https://doi.org/10.1073/pnas.82.13.4404.
16. Joglekar, A. P., and A. J. Hunt. 2002. A simple, mechanistic model for directional instability during mitotic chromosome movements. *Biophys. J.* 83:42–58.
17. Civelekoglu-Scholey, G., D. J. Sharp, …, J. M. Scholey. 2006. Model of chromosome motility in Drosophila embryos: adaptation of a general mechanism for rapid mitosis. *Biophys. J.* 90:3966–3982.
18. Gay, G., T. Courtheoux, …, Y. Gachet. 2012. A stochastic model of kinetochore–microtubule attachment accurately describes fission yeast chromosome segregation. *J. Cell Biol.* 196:757–774.
19. Civelekoglu-Scholey, G., B. He, …, D. Cimini. 2013. Dynamic bonds and polar ejection force distribution explain kinetochore oscillations in PtK1 cells. *J. Cell Biol.* 201:577–593.

20. Keener, J. P., and B. Shtylla. 2014. A mathematical model of force generation by flexible kinetochore-microtubule attachments. *Biophys. J.* 106:998–1007.

21. Banigan, E. J., K. K. Chiou, …, A. J. Liu. 2015. Minimal model for collective kinetochore–microtubule dynamics. *Proc. Natl. Acad. Sci. USA.* 112:12699–12704. https://doi.org/10.1073/pnas.1513512112.

22. Schwietert, F., and J. Kierfeld. 2020. Bistability and oscillations in cooperative microtubule and kinetochore dynamics in the mitotic spindle. *New J. Phys.* 22:053008. https://doi.org/10.1088/1367-2630/ab7ede.

23. Ciferri, C., S. Pasqualato, …, A. Musacchio. 2008. Implications for kinetochore-microtubule attachment from the structure of an engineered Ndc80 complex. *Cell.* 133:427–439.

24. Weissmann, F., G. Petzold, …, S. Westermann. 2016. biGBac enables rapid gene assembly for the expression of large multisubunit protein complexes. *Proc. Natl. Acad. Sci. USA.* 113:E2564–E2569. https://doi.org/10.1073/pnas.1604935113.

25. Fairhead, M., G. Veggiani, …, M. Howarth. 2014. SpyAvidin hubs enable precise and ultrastable orthogonal nanoassembly. *J. Am. Chem. Soc.* 136:12355–12363. https://doi.org/10.1021/ja505584f.

26. Tuszyński, J. A., T. Luchko, …, J. M. Dixon. 2005. Anisotropic elastic properties of microtubules. *Eur. Phys. J. E Soft Matter.* 17:29–35. https://doi.org/10.1140/epje/i2004-10102-5.

27. Marko, J. F., and E. D. Siggia. 1995. Stretching DNA. *Macromolecules.* 28:8759–8770.

28. Kierfeld, J., O. Niamploy, …, R. Lipowsky. 2004. Stretching of semiflexible polymers with elastic bonds. *Eur. Phys. J. E Soft Matter.* 14:17–34. https://doi.org/10.1140/epje/i2003-10089-3.

29. Pampaloni, F., G. Lattanzi, …, E.-L. Florin. 2006. Thermal fluctuations of grafted microtubules provide evidence of a length-dependent persistence length. *Proc. Natl. Acad. Sci. USA.* 103:10248–10253. https://doi.org/10.1073/pnas.0603931103.

30. Wei, R. R., J. Al-Bassam, and S. C. Harrison. 2007. The Ndc80/HEC1 complex is a contact point for kinetochore-microtubule attachment. *Nat. Struct. Mol. Biol.* 14:54–59.

31. Alushin, G. M., V. H. Ramey, …, E. Nogales. 2010. The Ndc80 kinetochore complex forms oligomeric arrays along microtubules. *Nature.* 467:805–810.

32. DeLuca, J. G., and A. Musacchio. 2012. Structural organization of the kinetochore–microtubule interface. *Curr. Opin. Cell Biol.* 24:48–56.

33. Flory, P. J. 1969. Statistical Mechanics of Chain Molecules. Interscience Publishers, New York.

34. McIntosh, J. R., E. O'Toole, …, N. Gudimchuk. 2018. Microtubules grow by the addition of bent guanosine triphosphate tubulin to the tips of curved protofilaments. *J. Cell Biol.* 217:2691–2708.

35. Oesterhelt, F., M. Rief, and H. E. Gaub. 1999. Single molecule force spectroscopy by AFM indicates helical structure of poly(ethylene-glycol) in water. *New J. Phys.* 1:6.1–6.11.

36. Huber, P. J. 1964. Robust estimation of a location parameter. *Ann. Math. Stat.* 35:73–101.

37. Pasche, S., M. Textor, …, H. J. Griesser. 2005. Relationship between interfacial forces measured by colloid-probe atomic force microscopy and protein resistance of poly(ethylene glycol)-grafted poly(l-lysine) adlayers on niobia surfaces. *Langmuir.* 21:6508–6520. https://doi.org/10.1021/la050386x.

38. Berg-Sørensen, K., and H. Flyvbjerg. 2004. Power spectrum analysis for optical tweezers. *Rev. Sci. Instrum.* 75:594–612. https://doi.org/10.1063/1.1645654.

39. Tolić-Nørrelykke, I. M., K. Berg-Sørensen, and H. Flyvbjerg. 2004. MatLab program for precision calibration of optical tweezers. *Comput. Phys. Commun.* 159:225–240.

40. Tegha-Dunghu, J., E. M. Gusnowski, and M. Srayko. 2014. An improved optical tweezers assay for measuring the force generation of single kinesin molecules. *Methods Mol. Biol.* 1136:103–116. https://doi.org/10.1007/978-1-4939-0329-0_7.

41. Thomas, W. E., V. Vogel, and E. Sokurenko. 2008. Biophysics of catch bonds. *Annu. Rev. Biophys.* 37:399–416. https://doi.org/10.1146/annurev.biophys.37.032807.125804.

42. Sarangapani, K. K., and C. L. Asbury. 2014. Catch and release: how do kinetochores hook the right microtubules during mitosis? *Trends Genet.* 30:150–159.

43. Liu, D., G. Vader, …, S. M. A. Lens. 2009. Sensing chromosome Bi-orientation by spatial separation of aurora B kinase from kinetochore substrates. *Science.* 323:1350–1353. https://doi.org/10.1126/science.1167000.

44. Caldas, G. V., K. F. DeLuca, and J. G. DeLuca. 2013. KNL1 facilitates phosphorylation of outer kinetochore proteins by promoting Aurora B kinase activity. *J. Cell Biol.* 203:957–969.

45. Yoo, T. Y., J.-M. Choi, …, D. J. Needleman. 2018. Measuring NDC80 binding reveals the molecular basis of tension-dependent kinetochore-microtubule attachments. *Elife.* 7:363922–e36434.

46. Akiyoshi, B., K. K. Sarangapani, …, S. Biggins. 2010. Tension directly stabilizes reconstituted kinetochore-microtubule attachments. *Nature.* 468:576–579.

47. Miller, M. P., C. L. Asbury, and S. Biggins. 2016. A TOG protein confers tension sensitivity to kinetochore-microtubule attachments. *Cell.* 165:1428–1439.

48. Scarborough, E. A., T. N. Davis, and C. L. Asbury. 2019. Tight bending of the Ndc80 complex provides intrinsic regulation of its binding to microtubules. *Elife.* 8:e44489.

49. Roscioli, E., T. E. Germanova, …, A. D. McAinsh. 2020. Ensemble-level organization of human kinetochores and evidence for distinct tension and attachment sensors. *Cell Rep.* 31:107535.

50. Sokurenko, E. V., V. Vogel, and W. E. Thomas. 2008. Catch-bond mechanism of force-enhanced adhesion: counterintuitive, elusive, but widespread? *Cell Host Microbe.* 4:314–323.

51. Schneider, S. W., S. Nuschele, …, M. F. Schneider. 2007. Shear-induced unfolding triggers adhesion of von Willebrand factor fibers. *Proc. Natl. Acad. Sci. USA.* 104:7899–7903. https://doi.org/10.1073/pnas.0608422104.

52. Interlandi, G., and W. Thomas. 2010. The catch bond mechanism between von Willebrand factor and platelet surface receptors investigated by molecular dynamics simulations. *Proteins.* 78:2506–2522. https://doi.org/10.1002/prot.22759.

53. Arbore, C., M. Sergides, …, M. Capitanio. 2021. α-catenin switches between a slip and an asymmetric catch bond with F-actin to cooperatively regulate cell junction fluidity. Preprint at bioRxiv. https://doi.org/10.1101/2020.04.15.035527.

54. Wimbish, R. T., and J. G. DeLuca. 2020. Hec1/Ndc80 tail domain function at the kinetochore-microtubule interface. *Front. Cell Dev. Biol.* 8:43–46. https://doi.org/10.3389/fcell.2020.00043/full.

55. Hvidt, S., J. D. Ferry, …, M. L. Greaser. 1983. Flexibility of light meromyosin and other coiled-coil α-helical proteins. *Macromolecules.* 16:740–745. https://doi.org/10.1021/ma00239a007.

56. Visscher, K., and S. M. Block. 1998. Versatile optical traps with feedback control. *Methods Enzymol.* 298:460–489.

57. Neuman, K. C., and S. M. Block. 2004. Optical trapping. *Rev. Sci. Instrum.* 75:2787–2809. https://doi.org/10.1063/1.1785844.

58. Sarshar, M., W. T. Wong, and B. Anvari. 2014. Comparative study of methods to calibrate the stiffness of a single-beam gradient-force optical tweezers over various laser trapping powers. *J. Biomed. Opt.* 19:115001.

59. Holland, P. W., and R. E. Welsch. 1977. Robust regression using iteratively reweighted least-squares. *Commun. Stat. Theor. Methods.* 6:813–827. https://doi.org/10.1080/03610927708827533.

60. de Menezes, D., D. Prata, …, J. Pinto. 2021. A review on robust M-estimators for regression analysis. *Comput. Chem. Eng.* 147:107254.

61. Casella, G., and R. L. Berger. 2002. Statistical Inference, 2 edition. Duxbury.

Biophysical Journal, Volume *121*

Supplemental information

Strain stiffening of Ndc80 complexes attached to microtubule plus ends

Felix Schwietert, Vladimir A. Volkov, Pim J. Huis in 't Veld, Marileen Dogterom, Andrea Musacchio, and Jan Kierfeld

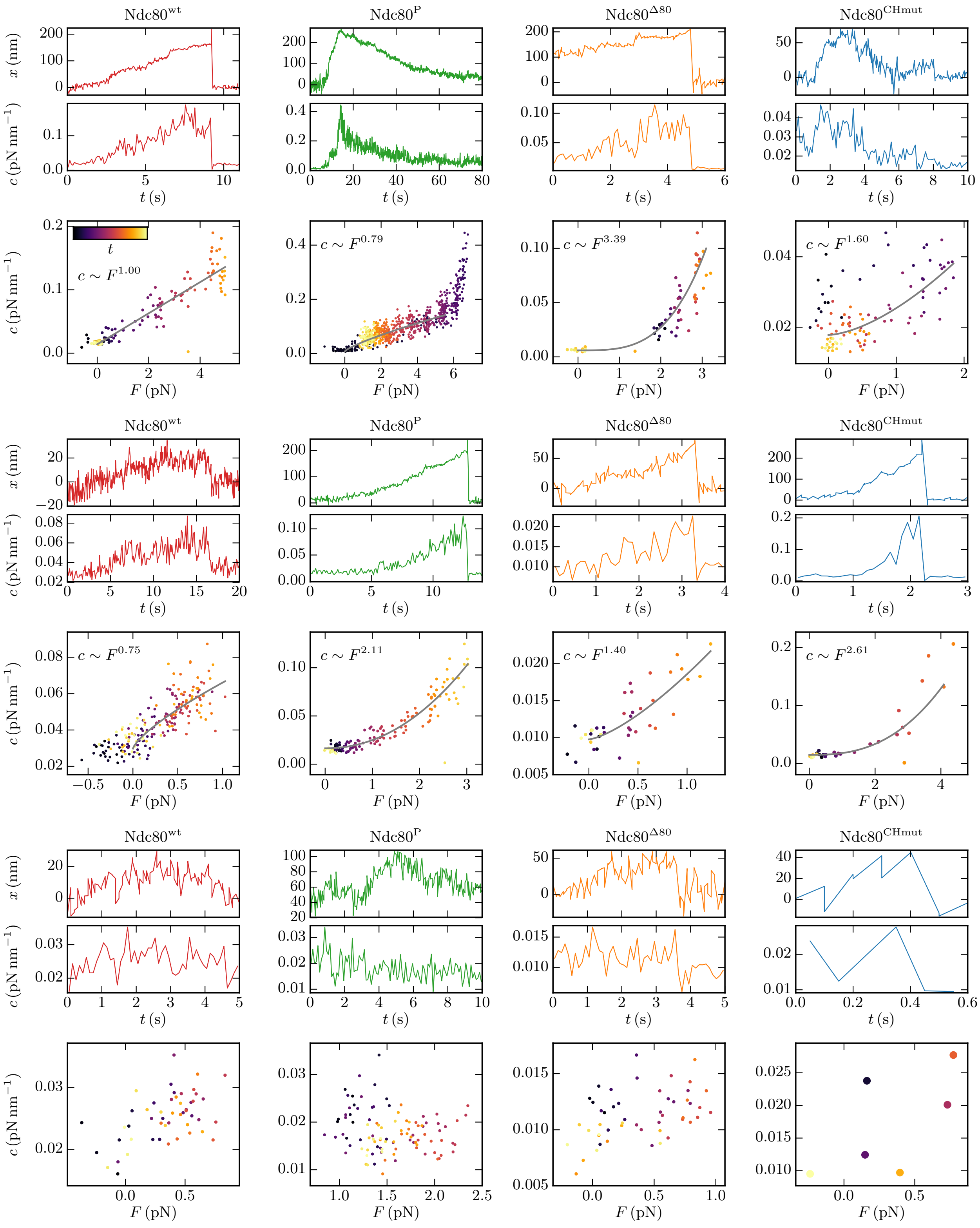

FIG. S1. Twelve more example experiments as described in Fig. 3A-D in the main text. The gray lines show powerfits to the stiffness–force relations that exhibit strain stiffening, giving the stiffening exponents depicted in Fig. 3F in the main text. The fits do not include values corresponding to $x > 200\,\mathrm{nm}$, see for instance the top Ndc80^{P} plot, where the fit only applies to $F \lesssim 6\,\mathrm{pN}$.

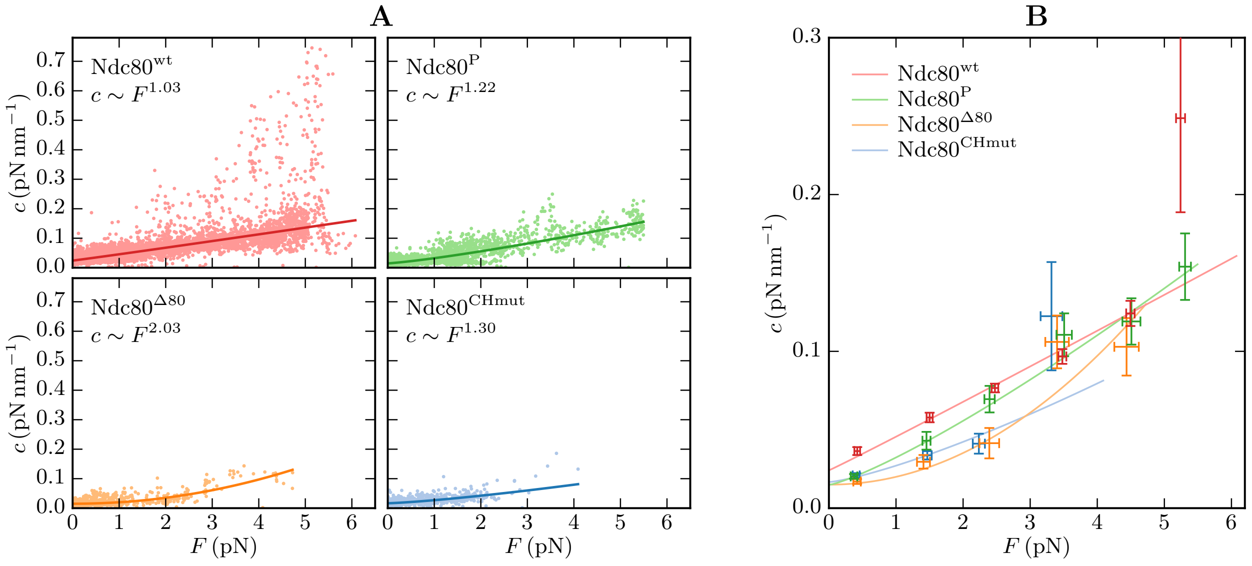

FIG. S2. Collective analysis including the experiments without observable stiffening. The main results are unchanged due to the comparably short durations of non-stiffening experiments. (A) Cumulative stiffness data with robust power fits analogously to Fig. 3F in the main text. The linear stiffening is maintained for Ndc80^{wt} and roughly for Ndc80^{P}. (B) Summarized stiffness–force relations analogously to Fig. 3G in the main text. The Ndc80^{wt} stiffness still exceeds the stiffnesses of Ndc80$^{\Delta 80}$ and Ndc80^{CHmut}.

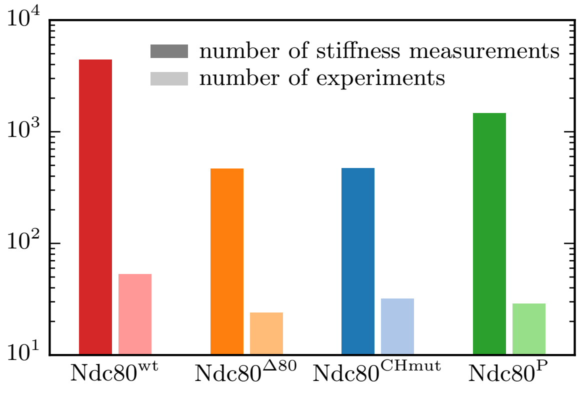

FIG. S3. Increased amount of stiffness data. While only one stiffness per experiment can be obtained by the sole use of the stalls for stiffness determination (right bars), the time tracing analysis increases the amount of data by 1 to 2 orders of magnitude (left bars).

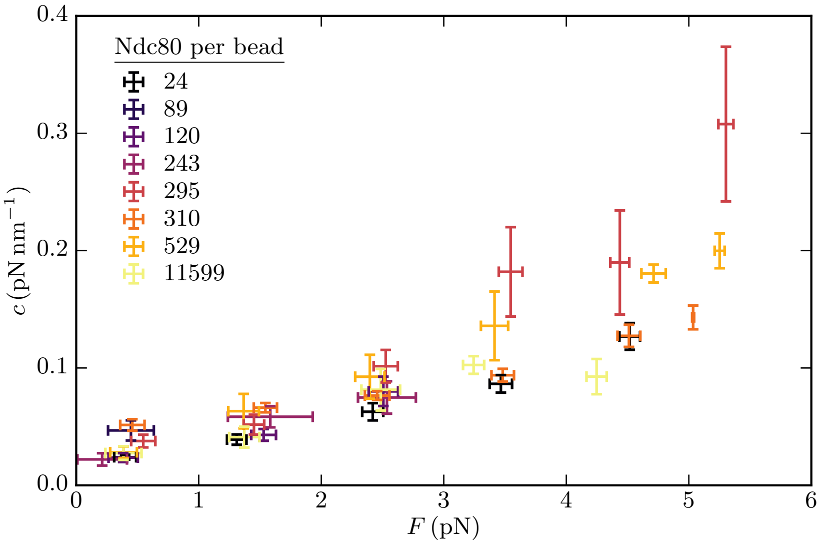

FIG. S4. Stiffness–force relations for different bead populations. The legend shows the mean number of Ndc80 complexes per bead in each population. There is no recognizable correlation between the stiffness and the Ndc80 density. The binning was performed as in Fig. 3H in the main text.

I. ROBUST FITTING TO THE STIFFNESS–FORCE RELATIONS

Here, we describe the robust fitting procedure of power law functions $c(F) = aF^b + c_0$ to the force–stiffness data (F_i,c_i). Since the stiffness values that result from the time tracing analysis exhibit a high spread (see Fig. 3E in the main text), we perform the power fits by use of a robust fitting method instead of a least squares fit which is sensitive to outliers. While in a least squares fit the sum of the squared residuals is minimized,

$$\min \frac{1}{2} \sum_i \epsilon_i^2, \quad \epsilon_i = \frac{c(F_i) - c_i}{\sigma_i}, \tag{S1}$$

the robust fitting method that we applied minimizes the sum of the Huber loss functions $\rho(\epsilon_i)$ [S1]:

$$\min \sum_i \rho(\epsilon_i), \quad \rho(\epsilon) = \begin{cases} \frac{1}{2}\epsilon^2, & |\epsilon| < k \\ k|\epsilon| - \frac{1}{2}k^2, & |\epsilon| \geq k \end{cases}. \tag{S2}$$

Thereby, the residuals are weighted as in a least squares fit for $\epsilon < k$, and with an absolute estimator (which results in a median when the data is fitted by a constant function) for $\epsilon \geq k$. The tuning parameter k is set to 1.345 to achieve a relative efficiency of 95 % in respect to the normal distribution [S2, S3]. Finally, we need to estimate the errors σ_i of the stiffnesses that we determined from the sample variances of the bead positions x within each interval (see Fig. 1 in the main text). Since $c \propto 1/\mathrm{Var}(x)$, the relative deviation of the the stiffness is the same as for the variance, $\sigma_c/c = \sigma_{\mathrm{Var}}/\mathrm{Var}(x)$. From a sample of length n, the variance and its deviation can be estimated to [S4]

$$\mathrm{Var}(x) = \frac{1}{n-1} \sum_i \left(x_i - \langle x \rangle\right)^2, \quad \sigma_{\mathrm{Var}} = \sqrt{\frac{2}{n-1}}\,\mathrm{Var}(x). \tag{S3}$$

With $n = 1000$ in a 0.1 s interval at a sample frequency of 10 kHz and stiffnesses around $c = 0.1\,\mathrm{pN\,nm^{-1}}$, the estimated error is

$$\sigma_c = \sqrt{\frac{2}{n-1}}\,c = 0.0045\,\mathrm{pN\,nm^{-1}}. \tag{S4}$$

The fit results and the original data are shown in Fig. 3E in the main text, the fit parameters are listed in Tab. S1.

TABLE S1. Fit parameters of power fits $c(F) = aF^b + c_0$.

	a	b	c_0
Ndc80^{wt}	0.022	1.03	0.025
Ndc80$^{\Delta 80}$	0.0038	2.21	0.017
Ndc80^{CHmut}	0.0069	2.00	0.017
Ndc80^{P}	0.027	0.98	0.010

II. MECHANICAL MT STIFFNESS

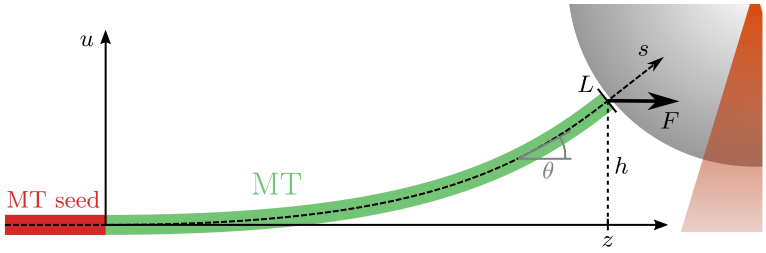

FIG. S5. Model for the effective stiffness from MT unbending.

The model for the mechanical stiffness that follows from MT unbending is similar to the PF model in the main text. In contrast to the PF, the MT does not have a spontaneous curvature but its tip is lifted to a height h by the attached bead as sketched in Fig. S5. With a force F that is applied in z-direction, the total energy is given by the sum of bending and stretching energies:

$$E_{\mathrm{MT}} = \int_0^L \left(\frac{\alpha}{2}(\dot\theta(s))^2 - F\cos\theta(s)\right) \mathrm{d}s, \tag{S5}$$

where s denotes the position along the MT, θ the local bending angle, L the length of the free MT end (behind the fixed MT seed), and $\alpha = k_{\mathrm{B}}TL_{\mathrm{p}}$ the bending stiffness. The configuration $\theta(s)$ that minimizes the energy under the constraint

$$h = \int_0^L \sin\theta(s)\,\mathrm{d}s \tag{S6}$$

satisfies the Euler-Lagrange equation

$$\ddot\theta = \frac{F}{\alpha}\sin\theta - \frac{F_h}{\alpha}\cos\theta \overset{\theta\ll 1}{\approx} \frac{F}{\alpha}\theta - \frac{F_h}{\alpha}, \tag{S7}$$

with a Largange-multiplier F_h, which corresponds to the force in u-direction that is necessary to hold the MT tip at height h. With the boundary conditions $\theta(0) = 0$ (due to the fixed MT seed) and $\dot\theta(L) = 0$, the approximated equation is solved by

$$\theta(s) = \frac{F_h}{F}\left(1 - \cosh(\lambda s) + \tanh(\lambda L)\sinh(\lambda s)\right), \quad \lambda := \sqrt{\frac{F}{\alpha}}, \tag{S8}$$

$$F_h = \frac{Fh/L}{1 - \frac{1}{\lambda L}\tanh(\lambda L)}. \tag{S9}$$

This results in the effective deflection

$$\begin{aligned} z &= \int_0^L \cos\theta(s)\,\mathrm{d}s \approx \int_0^L \left(1 - \frac{\theta^2(s)}{2}\right)\mathrm{d}s \\ &= L\left[1 - \frac{h^2}{4L^2}\left(\frac{3}{1-f(\lambda L)} - \left(\frac{\lambda L\, f(\lambda L)}{1-f(\lambda L)}\right)^2\right)\right], \quad f(x) := \frac{\tanh x}{x}. \end{aligned} \tag{S10}$$

Finally, the effective stiffness of MT unbending is given by

$$c = \left(\frac{\partial z}{\partial F}\right)^{-1}$$
$$= \frac{8\sqrt{F\alpha}}{h^2} \frac{\big(1 - f(\lambda L)\big)^3}{f'(\lambda L)\,\big((3 + 2\lambda^2 L^2)\, f(\lambda L) - 3\big) + 2\lambda L f^2(\lambda L)\,\big(1 - f(\lambda L)\big)}. \tag{S11}$$

In the limit of strong forces ($F \gg \alpha/L^2 \sim 0.05\,\mathrm{pN}$), this converges to

$$c \approx \frac{8L^2}{h^2\sqrt{\alpha}} F^{\frac{3}{2}}. \tag{S12}$$

There are two required parameters: the length L of the free MT end and the height h of the MT tip. Typical MT lengths lay around $L \sim 5\,\mathrm{\mu m}$. Given the bead diameter of $1\,\mathrm{\mu m}$ and the distance between the surfaces of the coverslip and the bead ($\sim 100\,\mathrm{nm}$), the height of the tip should not exceed $h \approx 500\,\mathrm{nm}$. Fig. S6 shows the mechanical MT stiffness c_{MT} compared to and in series with the stiffness of n parallel Ndc80 complexes for $L = 5\,\mathrm{\mu m}$ and $h = 500\,\mathrm{nm}$. Though this choice of h provides a lower estimate ($c_{\mathrm{MT}} \propto h^{-2}$), the mechanical MT stiffness is still too large, to have a significant influence.

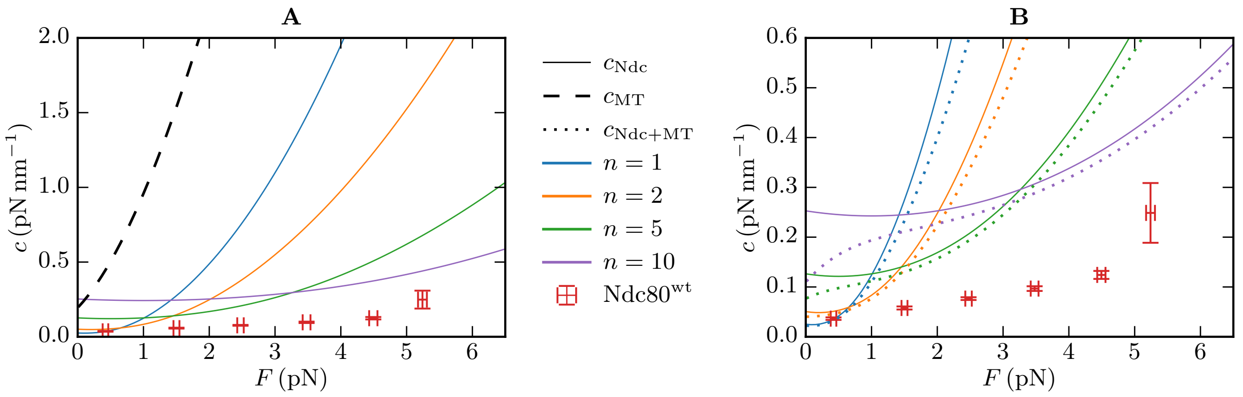

FIG. S6. (A) The mechanical stiffness of MT unbending (dashed line, $L = 5\,\mathrm{\mu m}$, $h = 500\,\mathrm{nm}$) exceeds the stiffness of n parallel Ndc80 complexes (solid lines) and the measured wild type stiffnesses. (B) The dotted lines show the mechanical MT stiffness in series with n Ndc80 complexes. The influence of the MT is not significant when $c_{\mathrm{Ndc}} \gg c_{\mathrm{MT}}$.

III. PEG STIFFNESS

According to Oesterhelt et al. [S5], the extension–force relation of PEG is given by:

$$L(F) = N_{\mathrm{S}} \left(\frac{L_{\mathrm{planar}}}{\mathrm{e}^{+\beta\Delta G(F)} + 1} + \frac{L_{\mathrm{helical}}}{\mathrm{e}^{-\beta\Delta G(F)} + 1} \right) \left(\coth(\beta F L_{\mathrm{K}}) - \frac{1}{\beta F L_{\mathrm{K}}} \right) + N_{\mathrm{S}} \frac{F}{K_{\mathrm{S}}}, \qquad \text{(S13)}$$

where $\Delta G(F) = \Delta G_0 - F\,(L_{\mathrm{planar}} - L_{\mathrm{helical}})$. The used parameters are listed in Tab. S2. Except N_{S}, they are taken from Ref. [S5]. Fig. S7 shows the stiffness $c_{\mathrm{PEG}}(F) = (\partial L(F)/\partial F)^{-1}$ compared to and in series with the Ndc80 stiffness.

TABLE S2. Parameters of the PEG model.

Description	Symbol	Value
number of segments	N_{S}	45
bond length of the planar (trans-trans-trans) conformation	L_{planar}	0.358 nm
bond length of the helical (trans-trans-gauche) conformation	L_{helical}	0.28 nm
Kuhn length	L_{K}	0.7 nm
zero force free energy difference $G_{\mathrm{planar}} - G_{\mathrm{helical}}$	ΔG_0	$3\,k_{\mathrm{B}}T$
segment elasticity	K_{S}	$1.5 \times 10^5\,\mathrm{pN\,nm^{-1}}$

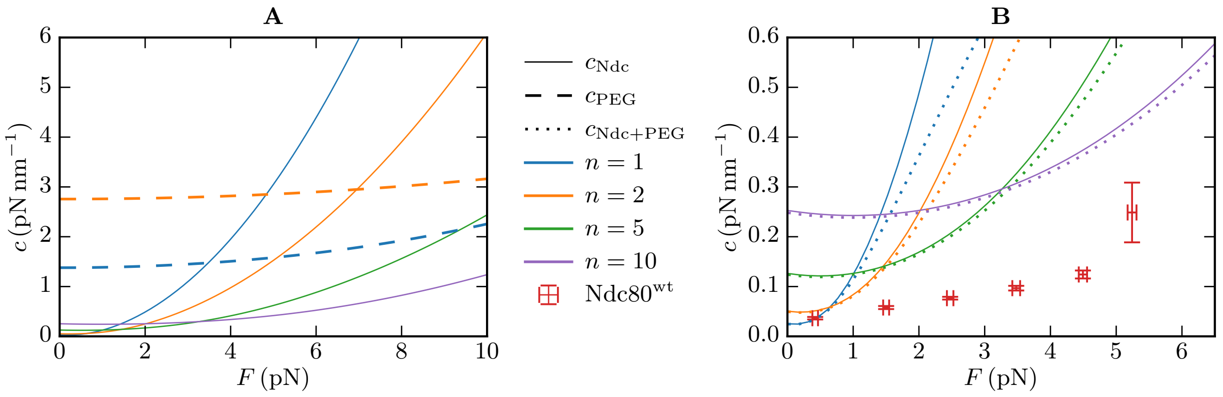

FIG. S7. (A) The stiffness of n parallel PEG molecules (dashed lines) exceeds the Ndc80 stiffness (solid lines) in most of the examined force range ($F < 6\,\mathrm{pN}$) and lies an order of magnitude above the measured stiffnesses ($\sim 0.1\,\mathrm{pN\,nm^{-1}}$). (B) The dotted lines show the stiffness of n PEG molecules in series with n Ndc80 complexes. The influence of PEG is not significant when $c_{\mathrm{Ndc}} \gg c_{\mathrm{PEG}}$.

IV. WORM-LIKE CHAIN MODEL

To take into account a Ndc80 complex with possibly (semi)flexible arms, we model the Ndc80 complex as two worm like chains (WLC) with persistence length L_p that are flexibly connected. To run Monte Carlo (MC) simulations, we describe each WLC by a bead–spring model [S6]: The Ndc80 arm $\vec{a}$ ($\vec{b}$) is discretized into $N_a + 1$ ($N_b + 1$) beads, which are connected by N_a (N_b) springs each with rest length d_a (d_b) and stiffness k_a (k_b). The rest lengths are defined by $d_a = a/N_a$ and $d_b = b/N_b$ to be consistent with the observed Ndc80 arm lengths a and b. Given the extensions and directions of the springs as $\vec{a}_i$ ($\vec{b}_j$) with $i = 1...N_a$ ($j = 1...N_b$), we find the total stretching energy

$$E_\mathrm{stretch} = \frac{k_a}{2}\sum_{i=1}^{N_a}\left(|\vec{a}_i| - d_a\right)^2 + \frac{k_b}{2}\sum_{j=1}^{N_b}\left(|\vec{b}_j| - d_b\right)^2. \tag{S14}$$

The positions $\vec{A}_m$ and $\vec{B}_n$ of the beads are given by

$$\vec{A}_m = \vec{A}_0 + \sum_{i=1}^{m}\vec{a}_i, \qquad \vec{B}_n = \vec{B}_0 + \sum_{j=1}^{n}\vec{b}_j, \tag{S15}$$

where $\vec{A}_0 = \vec{0}$ (the Ndc80 complex is fixed to the glass bead) and $\vec{B}_0 = \vec{A}_{N_a}$. The glass bead, which is modeled as a wall (see Fig. 2C in the main text), is described by the boundary condition that each bead has to be located in the upper half space, i.e., for the z-components: $A_{m,z}, B_{n,z} > 0$ for each m, n. Each bead $\vec{A}_m, \vec{B}_n$ except $\vec{A}_0$, $\vec{A}_{N_a} = \vec{B}_0$ and $\vec{B}_{N_b}$ contributes a bending energy which sums up to

$$E_\mathrm{bend} = \frac{L_\mathrm{p}}{\beta d_a}\sum_{m=1}^{N_a-1}(1 - \cos\alpha_m) + \frac{L_\mathrm{p}}{\beta d_b}\sum_{n=1}^{N_b-1}(1 - \cos\beta_n), \tag{S16}$$

where α_m (β_n) is the angle between bonds m and $m+1$ (n and $n+1$):

$$\cos\alpha_m = \frac{\vec{a}_m \cdot \vec{a}_{m+1}}{|\vec{a}_m||\vec{a}_{m+1}|}, \quad \cos\beta_n = \frac{\vec{b}_n \cdot \vec{b}_{n+1}}{|\vec{b}_n||\vec{b}_{n+1}|}. \tag{S17}$$

Finally, when a force F in z-direction is applied on the last bead, the total energy reads as:

$$E = E_\mathrm{stretch} + E_\mathrm{bend} - FB_{N_b,z}. \tag{S18}$$

In the MC simulation, we randomly choose a bead ($i, j > 0$) and suggest to move it in a random direction for a constant distance s. If the move does not violate the boundary condition, it will be accepted with probability $\min(1, \exp(-\beta\Delta E))$, where $\Delta E = E_\mathrm{sugg} - E_\mathrm{orig}$

is the energy difference between the suggested and the original configuration following Eq. (S18). After a certain time of equilibration, we measure $z = B_{N_b,z}$ and z^2 after each sweep ($N_a + N_b$ moves), and calculate the mean extension and its variance at the end of a simulation. By repeating it for various external forces, we can record force–extension and force–stiffness relations.

In our simulations, we used $k_a = k_b = 1000\,\mathrm{pN\,nm^{-1}}$ to model the two Ndc80 arms as nearly unstretchable. Moreover, we used the same discretization lengths $d_a = d_b = 1\,\mathrm{nm}$, i.e., $N_a = 40$ and $N_b = 16$.

Fig. S8 shows the stiffness–force relations for a single and for $n = 5$ parallel semiflexible Ndc80 complexes with various persistence lengths L_p. The effect of semiflexibility on the stiffness is negligible for realistic persistence lengths above 100 nm [S7].

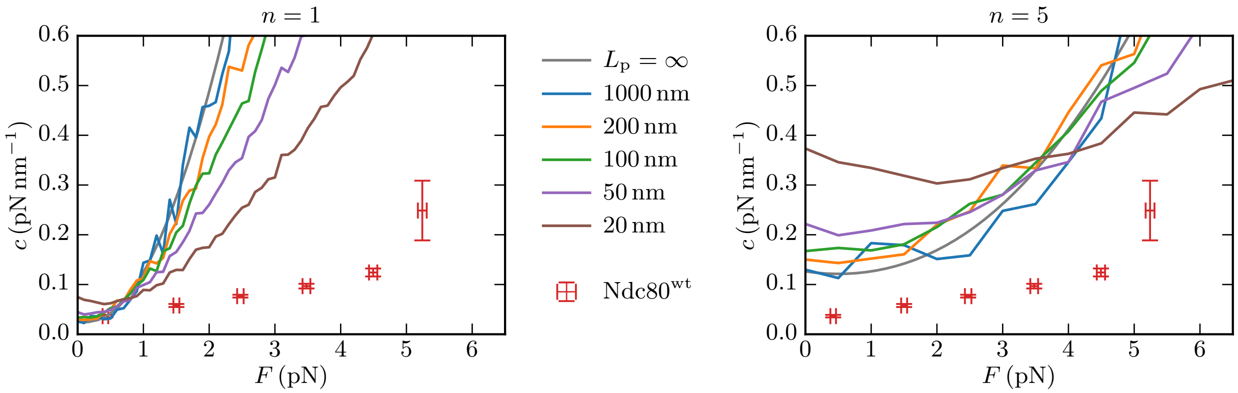

FIG. S8. Stiffness of a single (left) and $n = 5$ parallel (right) semiflexible Ndc80 complexes with persistence lengths L_p. As a check of the MC simulations, we added the large persistence length $L_\mathrm{p} = 1000\,\mathrm{nm}$, which correctly resembles results of the FJC model ($L_\mathrm{p} = \infty$) according to Eq. (8) in the main text.

SUPPORTING REFERENCES

[S1] Huber, P. J., 1964. Robust Estimation of a Location Parameter. *Ann. Math. Stat.* 35:73–101. http://projecteuclid.org/euclid.aoms/1177703732.

[S2] Holland, P. W., and R. E. Welsch, 1977. Robust regression using iteratively reweighted least-squares. *Commun. Stat. - Theory Methods* 6:813–827. http://www.tandfonline.com/doi/abs/10.1080/03610927708827533.

[S3] de Menezes, D., D. Prata, A. Secchi, and J. Pinto, 2021. A review on robust M-estimators for regression analysis. *Comput. Chem. Eng.* 147:107254. https://linkinghub.elsevier.com/retrieve/pii/S0098135421000326.

[S4] Casella, G., and R. L. Berger, 2002. Statistical inference. Duxbury, Pacific Grove, CA 93950 USA, 2 edition.

[S5] Oesterhelt, F., M. Rief, and H. E. Gaub, 1999. Single molecule force spectroscopy by AFM indicates helical structure of poly(ethylene-glycol) in water. *New J. Phys.* 1:6.1–6.11. https://iopscience.iop.org/article/10.1088/1367-2630/1/1/006.

[S6] Kierfeld, J., O. Niamploy, V. Sa-yakanit, and R. Lipowsky, 2004. Stretching of semiflexible polymers with elastic bonds. *Eur. Phys. J. E* 14:17–34. http://link.springer.com/10.1140/epje/i2003-10089-3.

[S7] Hvidt, S., J. D. Ferry, D. L. Roelke, and M. L. Greaser, 1983. Flexibility of light meromyosin and other coiled-coil α-helical proteins. *Macromolecules* 16:740–745. https://pubs.acs.org/doi/abs/10.1021/ma00239a007.
